# A striking difference: biomechanics of the impaling hunting strategy of a moss mantis

**DOI:** 10.1186/s12983-026-00610-9

**Published:** 2026-04-11

**Authors:** Fabian Bäumler, Stanislav N. Gorb, Sebastian Büsse

**Affiliations:** 1 Functional Morphology and Biomechanics, Institute of Zoology, Kiel University, Kiel, Germany; 2 Cytology and Evolutionary Biology, Institute of Zoology and Museum, University of Greifswald, Greifswald, Germany

**Keywords:** Praying mantis, Raptorial forelegs, Latch-mediated spring actuation, Prothoracic legs, Prey capture, Ecomorphology, *Haania orlovi*

## Abstract

**Background:**

Biological catapults as power amplification systems are widespread across diverse taxa, known for their evolutionary significance and effectiveness in various ecological contexts. Although praying mantises are renowned for their predatory behavior, typically involving a directly muscle-driven, grasping-like motion to capture prey, a strongly altered movement sequence is observed in *Haania orlovi*. The moss mantis exhibits a spear-like foreleg morphology and a significantly different ultrafast impaling hunting strategy.

**Results:**

This system generates a mass-specific power output, surpassing the limits of direct muscle contraction. Through comprehensive morphological analysis (micro-computed tomography, scanning electron microscopy), combined with high-speed videography and force measurements, we provide evidence for a latch-mediated spring actuation (*LaMSA*) system, enabling this ballistic motion. The mechanism involves elastic energy storage in the deformed cuticle of the proximal trochanter, supported by latch-like interlocking. Confocal laser scanning microscopy revealed specialized cuticle composition in the trochanter, facilitating energy storage. For further validation, we developed a 3D-printed proof-of-concept model, incorporating a deformable spring-like double-spiral structure, demonstrating the functional advantage of the *LaMSA* system in generating high-speed movements.

**Conclusion:**

This study not only elucidates a novel predatory mechanism in mantises but also contributes to our understanding of evolutionary adaptations in predator–prey interactions. Illustrating the essential mechanical components in a physical model, and the compact, load-responsive dual functionality of the described power amplification system, potentially serves as inspiration for advancements in bio-inspired engineering solutions. Our findings highlight the importance of integrated biomechanical analysis in uncovering novel biomechanical mechanisms, demonstrating potential for significant functional shifts through seemingly minor morphological modifications.

**Supplementary Information:**

The online version contains supplementary material available at 10.1186/s12983-026-00610-9.

## Background

Fast movements are crucial in many biological processes, particularly in predator–prey interactions and locomotion. These rapid movements allow especially small organisms, to capture elusive prey, escape predators, or navigate challenging environments with remarkable speed and precision [1]. To accomplish such fast movements in animals, the muscles associated with the motion must generate a very high mechanical power output (*mpo*). However, in several groups, the observed movement velocities exceed the limits of direct muscle contraction. The rate at which a muscle can perform work: its *mpo*—depends on the force the muscle can exert and the rate at which it can shorten [2]. However, the *mpo* of a muscle alone is limited and can either be optimized to generate high forces or achieve a high contraction speed, but not both simultaneously [3–6]. Thus, these movements employ specialized anatomical structures and biomechanical mechanisms, often involving elastic energy storage and rapid release through latch-mediated spring actuation (*LaMSA*) systems, combined with optimized musculoskeletal gearing, optimizing the muscles’ energy output to overcome these limitations [6–8]. These so-called biological catapults enable animals to achieve speeds and acceleration far beyond those provided by muscle power alone [5]. These catapult mechanisms are widespread in nature, extending beyond the animal kingdom [1, 9–14], allowing organisms to rapidly release stored elastic energy to generate explosive movements [6, 7, 15, 16]. The prevalence of these mechanisms across diverse taxa highlights their evolutionary significance and effectiveness in various ecological contexts [7, 16, 17].

**Fig. 1:**
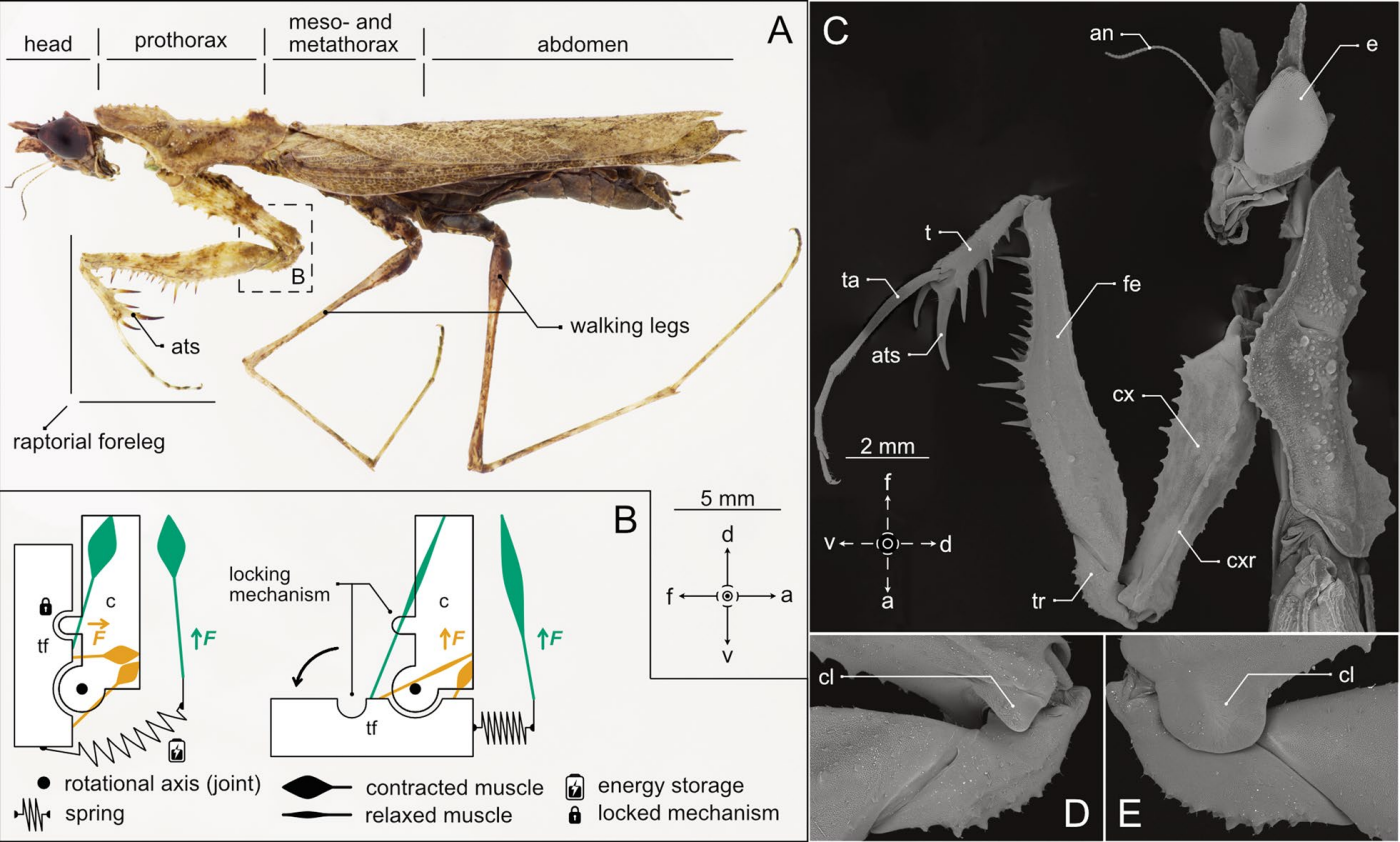
Overview of *H. orlovi* and its prey-impaling principle. **A**, **B** Female *H. orlovi* in lateral view (**A**), with a simplified scheme of the functional principle of the *LaMSA* system in (**B**). **C** Shows an SEM overview image of *H. orlovi* in lateral view, with an additional close-up of the trochanter in posterior (**D**) and anterior (**E**) view. Abbreviations: a—abdominal; an—antenna; ats—anterior tibial spine; cx—coxa; cl—coxal lobe; cxr—coxal ridge; d—dorsal; e—eye; fe—femur; F—force; f—frontal; ta—tarsus; t—tibia; tf—trochanter-femur; tr—trochanter; v—ventral

**Fig. 2:**
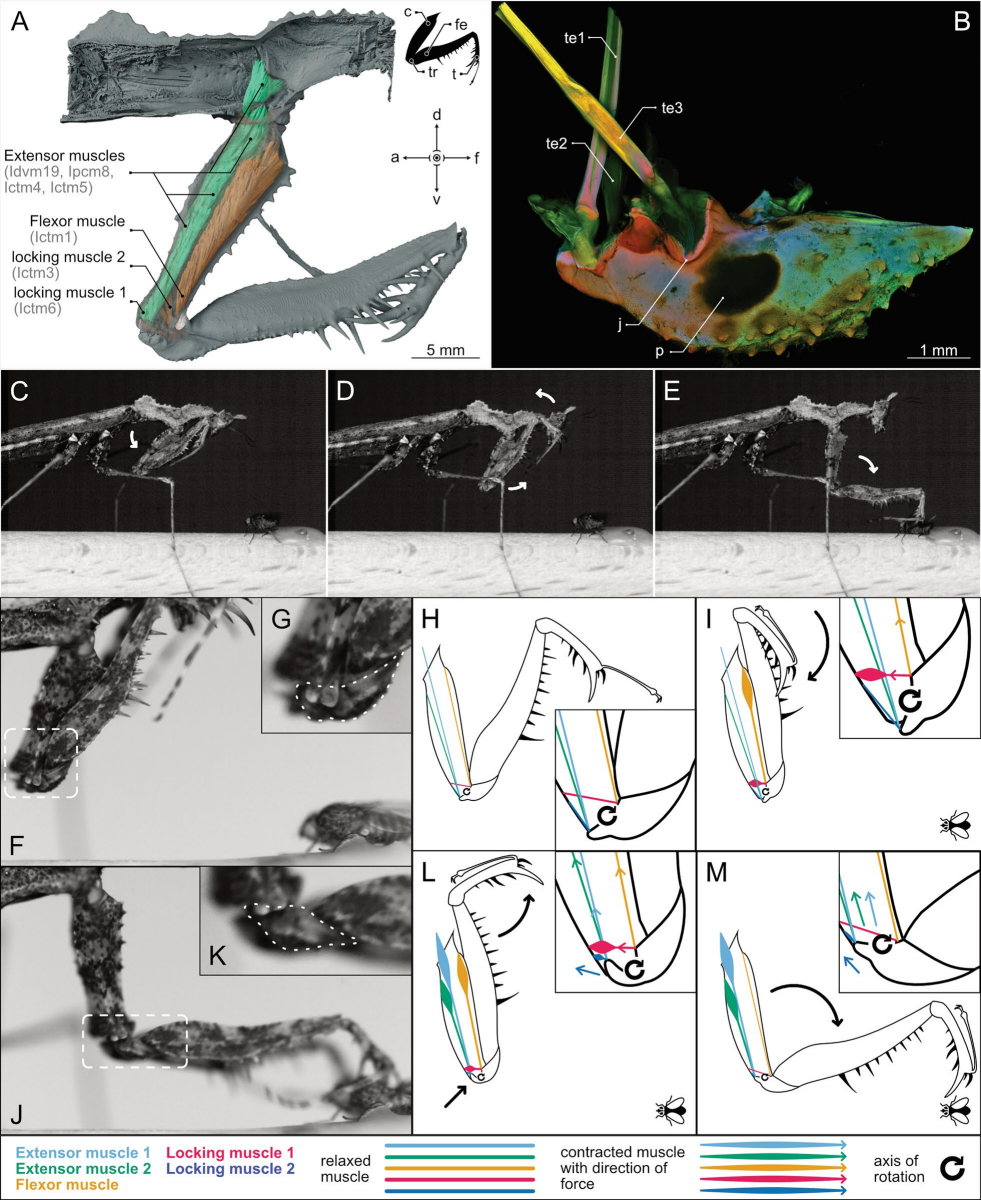
Three-dimensional visualization of the skeleton-muscle organization from µCT data and a scheme, as well as images from video footage, showing the important steps of the spearing motion of the predatory foreleg in *H. orlovi*. **A** The musculature of the raptorial foreleg and the prothorax, associated with the *LaMSA* in medial view. Muscles are grouped by function with their corresponding names written within light-grey brackets. In certain areas, the cuticle is displayed transparently to allow for a better view of the musculature. **B** A *CLSM* image of the dissected trochanter and the associated tendons in anterior view. **C**–**E** The movement sequence was obtained from high-speed videography footage (the associated high-speed video can be seen in Movie S1). **C** Approaching phase. **D** Preloading of the *LaMSA*. **E** Activation of the *LaMSA* and impaling of the prey item. **F**, **G**, **J**, **K** Detailed view of the deformation of the trochanter during the preloading process (highlighted by a white, dashed line; the associated video can be seen in Movie S2). **H**, **I**, **L**, **M** A scheme of the proposed striking mechanism. **H** Relaxed state of the system. **I** The moment the animal has noticed the prey item. **L** The preloading process with the deformed trochanter. **M** The final *LaMSA* release. A legend for the different colors and line styles can be found in the bottom of the figure. The direction of force is displayed by arrows, in some cases next to the related muscle in the same color for clarity. Abbreviations: a—abdominal; c—coxa; ctm—coxo-trochanteral muscle; d—dorsal; dvm—dorso-ventral muscle; fe—femur; f—frontal; j—joint; p—pigmented area; pcm—pleuro-coxal muscle; te—tendon; t—tibia; tr—trochanter; v—ventral

**Fig. 3:**
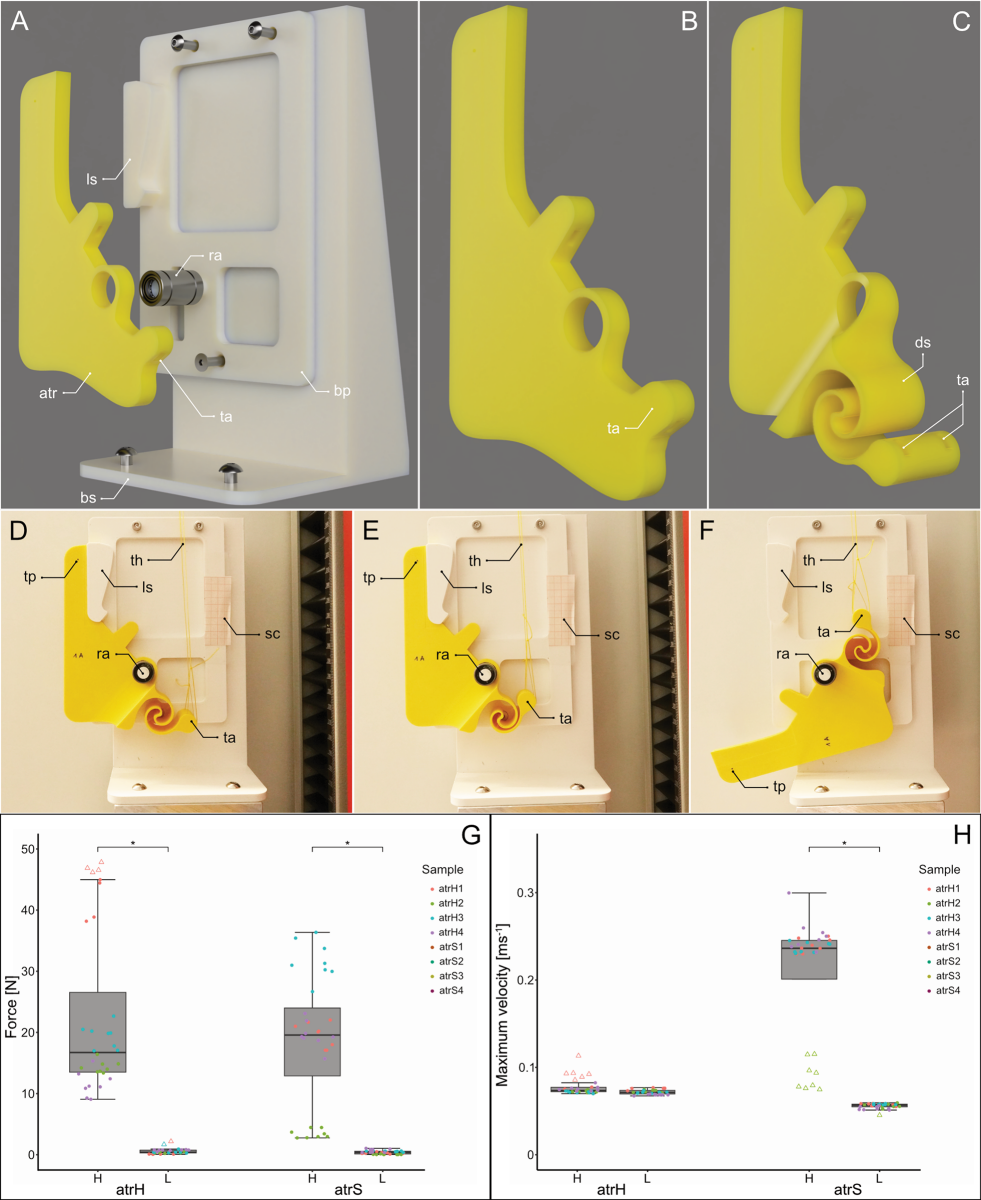
The 3D-printed proof-of-concept model of the *LaMSA* of *H. orlovi*. **A** Rendered images of the 3D-printed model with a non-deformable trochanter. **B** and **C** The non-deformable and deformable trochanter (latter with the double spiral spring). **D**–**F** Steps of the testing sequence from video footage, using a deformable trochanter. **D** The beginning of the test. **E** Maximum tensile load is achieved (double spiral spring is deformed), when the trochanter loses its contact to the locking structure. **F** The end of the movement, after the trochanter swung free. **G** A Boxplot of the measured forces. **H** A Boxplot of the calculated maximum angular velocities. Both latter plots show results for both artificial trochanters (*atrH*—stiff and *atrS*—flexible) and both experimental scenarios: for the higher friction (**H**) (*N* = 4 per trochanter type, *n* = 8 per testing object) and for the lower friction (**L**) (*N* = 4 per trochanter type,* n* = 8 per testing object). Individual datapoints are displayed in a color code to differentiate between samples. The upper and lower borders of the boxes indicate the 25th and 75th percentiles, the line within the box represents the median, and the whiskers indicate the 10th and 90th percentiles. Outliers are represented by black dots. The asterisk indicates statistically significant differences: * ≙ *p* < 0.001. Abbreviations: atr—artificial trochanter; atrH—artificial trochanter stiff; atrS—artificial trochanter flexible; bp—baseplate; bs—base structure; ds—double spiral; H—high friction scenario; L—low friction scenario; ls—locking structure; pf—platform; ra—rotational axis; sc—scale; ta—threat attachment; th—threat; tp—tracking point

Due to their often small size, especially in many groups of invertebrates, tendons and similar structures are employed as power amplifiers, leading to a variety of different implementations [5, 7, 18]. One of the most famous examples of ultrafast motions in biology are Stomatopoda (mantis shrimps), which employ a biological catapult for prey capture [19–21]. Mantis shrimps utilize a saddle-shaped spring mechanism, storing elastic energy, to generate incredibly fast strikes for hunting prey, accelerating their raptorial appendages to up to 23 ms^−1^—ranking among the fastest movements in the animal kingdom [19–24]. This remarkable speed allows mantis shrimps to crack open hard-shelled prey [25], and even inflict serious damage on prey items and rivals in territorial conflicts [26]. Similar motion sequences can also be observed in some representatives of Mantodea (praying mantises) [27]. This order of insects is adapted to its environment and role as an arthropod predator, both behaviorally and morphologically, using its prehensile raptorial forelegs as predatory grasping devices [28–38]. Although the predatory strike of mantodeans [31, 34, 38–40] is generally known as a directly muscle-driven grasping motion [41], members of a small group within Mantodea feature strong morphological interspecific variations and a vast modification of the prey capturing behavior [27, 42]. Strongly deviating from the previously mentioned grasping motion in the majority of mantodeans [27, 43, 44], the herein presented transformed raptorial forelegs of *H. orlovi* are moved in a ballistic high-speed motion, utilizing the spear-like reinforced spines of the tibia (Fig. 1A, C), impaling their prey item with velocities of ~ 5 ms^−1^. We present strong evidence of a power amplification, enabling the high mass-specific *mpo* of approximately 30,000–60,000 Wkg^−1^ of this impaling motion, which is shown in a simplified scheme in Fig. 1B. The system is mainly based on elastic energy storage in the deformed cuticle of the proximal region of the trochanter caused by the co-activated contraction of the large extensor and flexor muscles of the coxa (Figs. 1B, 2L). This is further supported by a latch-like cuticle interlocking of the distal coxal protrusions (Fig. 1D, E) with corresponding dents of the trochanter (Fig. S3), stabilized through the contraction of locking muscles 1 and 2 (Fig. 2L). In our study, we used experimental force measurements to avoid an overestimation of the *LaMSA* system and account for the *mpo* of the possible optimization of the entire gearing system [8]. Our results strongly indicate that the *mpo* required to achieve the observed angular velocities and forces far exceeds what the associated musculature can generate through direct contraction alone. Based on established principles of muscle action [3–5, 45], this result supports the presence of an elastic power amplification. To further support this statement and to test the mechanical feasibility of the proposed *LaMSA* mechanism, we designed a biologically-inspired, 3D-printed proof-of-concept model, isolating the core geometric components, the underlying elastic energy storage and release mechanisms (Fig. 3A). Such physical models are a well-established tool commonly used in biomechanics to illustrate biological movement principles by reducing complex biological systems to their essential mechanical components, offering a different perspective on the process [46–48]. Combining organismal studies and physical models is an effective way to advance form-function research, as it offers the advantage of controlled manipulation of variables and parameters which is otherwise experimentally impractical or even impossible [48]. Working with the design in an enlarged and simplified way proved extremely helpful in facilitating the understanding of the investigated mechanism in this study. Manufacturing the models using 3D-printing allows for controlled variation of geometry and material properties, and enables the production of several nearly identical samples for subsequent testing [48–50]. In a very simplified approach, we show the impact and resulting advantage of the presence of a *LaMSA* in the described mechanical system. The deformability of the artificial trochanter, that is simulating the energy storage in the preloading process, is achieved by adding a deformable double-spiral structure generated by using the Double-spiral design software [51]. The double spiral shape was chosen because of its potential to undergo predictable deformations and act like a spring, while having the possibility to directly incorporate it in our design process of the artificial trochanter and manufacturing it in one coherent piece [51, 52].

The aforementioned impaling hunting behavior in Mantodea is assumed to yield advantages while hunting small prey, especially in structurally complex habitats, which would otherwise render a grasping hunting strategy unfeasible (e.g., crevices the praying mantis has no direct access to) [27]. Mantises were observed to specifically target minute arthropods, even though they demonstrated the ability to successfully hunt larger prey, like blow flies [27]. This indicates an adaptation toward smaller-sized prey items, which would most likely have been ignored by mantises of similar size using grasping hunting behavior [27]. The convergent evolution of these described biological power amplification systems across different taxa underscores their effectiveness and adaptability to various ecological niches and functional requirements. Understanding the principles behind such high-speed biological movements provides insights into evolutionary adaptations, contributing to our general understanding of such systems in evolutionary biology. It showcases how similar physical principles can be exploited by diverse organisms to solve comparable functional challenges. Research on the biomechanics of high-speed movements provides valuable insights into biological systems, and in some cases, inspired new technological innovations or led to advancements in existing ones, as was shown in the past. For instance, the studies of mantis shrimp appendages have led to the development of new impact-resistant materials [53, 54], while the mechanics of trap-jaw ant mandibles have inspired the design of fast-acting robotic systems [6, 55–57]. The herein-presented combination of elastic energy storage and physical interlocking in praying mantises represents a simple, yet compact biological power amplification system.

## Methods

### Animals

Female specimens of the praying mantis species *H. orlovi* anisyutkin 2005 were obtained from private breeders in early larval stages and raised to adulthood, as well as bred in Kiel. All animals were kept individually in 19 × 19 × 19 cm “BraPlast” boxes (Terraristik Hörnchen GmbH, Bergheim, Germany), that were misted several times a week to achieve a humidity of approximately 60–70%, with temperatures ranging from 21 °C at night to 27 °C during the daytime and a light:dark regime of 12:12 h. BraPlast boxes featured mesh ventilation areas (black pollen protection fabric, on the lid and back of the box). Mantises were fed twice a week with *Drosophila melanogaster* meigen 1830, in earlier larval stages, and in later stages with *Drosophila hydei* sturtevant 1921 and *Thermobia domestica* (packard 1873).

### Photography

Overview imaging and stacked photography of the animal (Fig. 1) in a 3D-printed illumination dome system [58] were performed using an Olympus OMD 10mkII digital camera (Olympus K.K., Tokyo, Japan) in combination with a Leica 45 mm macro lens (Leica Camera AG, Wetzlar, Germany). Subsequent post-processing and labeling was done using Affinity Designer 2 and Affinity Photo 2 (Serif (Europe) Ltd., Nottingham, United Kingdom).

### Description of morphological investigation

For all morphological descriptions, the forelegs are characterized under the assumption that they are oriented in the way typical walking legs are, as proposed by Wieland [36], using the updated nomenclature introduced by Brannoch et al. [30]. In the descriptions, the non-moving end of each muscle is called the point of origin (O) and its moving end the point of insertion (I). Additional important information is listed in characteristics (C). Extrinsic muscle names follow the nomenclature introduced by Friedrich and Beutel [59], intrinsic muscles are named similarly and follow the nomenclature introduced by Bäumler and colleagues [29]. Cuticle structures were described using the updated nomenclature introduced by Brannoch et al. [30], supplemented by the works of Snodgrass [60], Gray and Mill [61], Friedrich and Beutel [59] and Wieland [36]. For an overview of the internal sclerites and cuticular structures, please refer to Fig. 3 in Bäumler et al. [28] and Fig. 2 in Bäumler et al. [29].

### Micro-computed tomography (µCT)

For µCT analysis, specimens were cooled and subsequently euthanized with CO_2_ and then fixed in Bouin’s solution (= Duboscq-Brasil [62]). The samples were washed and stored in 70% ethanol. Before scanning, samples were dehydrated in an ascending ethanol series (70, 80, 90, 95, and 99%) and subsequently dried using a fully automatic critical point dryer (Leica EM CPD300, Leica, Wetzlar, Germany). To dampen vibrations, the dried specimens were embedded in semi-soft shoe cleaning foam (*domol* Schmutzradierer, Rossmann home brand domol, Dirk Rossmann GmbH, Burgwedel, Germany). Scans were performed with a Skyscan® 1172 micro-CT scanner (Bruker micro-CT, Kontich, Belgium) with the following settings: X-ray source: 40 kV, 250 µA, 360° rotation, 0,2 rotation step, 10 frame averaging, and 10 random movements; image pixel size: 2,28 µm. The resulting images were subsequently reconstructed using Nrecon® 1.0.7.4 (Bruker micro-CT, Kontich, Belgium), processed in Amira® 6.2 (Thermo Fisher Scientific, Waltham, Massachusetts, USA), and finally visualized using the open-source 3D creation suite Blender 4.3.0 LTS (Blender Foundation, Amsterdam, Netherlands) and Affinity Designer 2 (Serif (Europe) Ltd., Nottingham, United Kingdom). For further validation, additional specimens were frozen at -70 °C for dissection to verify the muscle attachment points. Dissection was performed using a stereomicroscope and dissecting instruments.

### Scanning electron microscopy (SEM)

A freshly killed specimen of *H. orlovi* was mounted and subsequently air-dried. The specimen was then fixed to a toothpick, mounted on a SEM-stub, and sputter-coated with a 10 nm layer of gold–palladium (Leica Bal-TEC SCD500). SEM images were obtained using a Hitachi TM3000 (Hitachi High-Technologies Corp., Tokyo, Japan) at 15 kV acceleration voltage on a rotatable specimen holder [63] (Fig. 3A–E). All images (Fig. 1C–E) were post-processed using Affinity Photo 2 and Affinity Designer 2 (Serif (Europe) Ltd., Nottingham, United Kingdom).

### High-speed videography (HSV)

High-speed videos for motion analysis were obtained at the same temperature the mantises were kept at, using a Photron FASTCAM SA1.1 high-speed video camera (model 675 K-M1; Photron, Tokyo, Japan) with a 105 mm/1:2.8 macro lens (Sigma, Tokyo, Japan), mounted on a Manfrotto 055 tripod with a Manfrotto 410 geared head (Manfrotto, Spa, Italy), in combination with two Rotolight AEOS light sources (at 100%, 4500 K; Appleworld UK Ltd., Iver Heath, United Kingdom). A schematic of the experimental setup is shown in Fig. S1A. For the experiment, using molten wax, a fruit fly (*D. hydei*) was attached to a wooden stick aligned perpendicularly to the camera view. Mantises were then placed on the upper side of the stick at a distance, allowing it a natural approach to choose its optimum striking distance. For the length measurements, a piece of graph paper was glued to the stick. All recordings were taken at 12,000–20000 fps and stored as a sequence of single “.*tif*” images using the PFV 318 FASTCAM Viewer Software (Photron, Tokyo, Japan). Videos of 13 animals were obtained, with 1–6 videos per animal (Table S1).

### Motion tracking

The obtained high-speed video footage was analyzed using the visual effects software Adobe after Effects CS6 (Adobe Systems Software, San José, CA, USA) to obtain frame-by-frame information of the mantis body and forelimbs during the strike, using the software’s “Motion Tracking” workspace and its tracking algorithm. Five different structures were tracked during this process: (1) the body at the base of the forewings (blue), (2) the coxal base (green), (3) the coxa-trochanter joint (orange), (4) the femur-tibia joint (purple) and (5) the base of the tarsus (mustard; Fig. S2). The X and Y coordinates of each tracker were extracted as a “.*txt*” file for subsequent processing and statistical analysis using a JavaScript plugin from Koehnsen et al. [64]. Using R, vectors between the previously described points were obtained, and parameters (duration, angular velocity etc.) of the associated joint angles between these vectors were determined for all frames: coxa-trochanter-femur CT angle, femur-tibia FT angle, and the tip of the tibia. The initial angle for every joint was obtained from the raw angles at time point 0 (the first frame of the recording). From these data, standardized angles were obtained for all the joint angles by subtracting the original angle from the initial angle for each joint. The start of the impaling motion will be defined as the moment when the CT joint starts to increase, after it has been completely closed. Finally, the strike ends with the prey item being impaled (ats stopping to further penetrate the prey item). All kinematic data used for calculations of the *mpo* are shown in Table S1.

### Force measurements

Force measurements were performed at the same room temperature at which the mantises were kept at. A schematic and a photograph of the experimental setup are shown in Fig. S1B. A 25 g force transducer (Force transducer 25 g capacity, FORT25, World Precision Instruments, Sarasota) was used for the experiments, secured within the 3D-printed experimental setup in a horizontal position, and connected to a BIOPAC MP 100 data acquisition unit with a BIOPAC TCI-102 System (BIOPAC Systems, Inc., Goleta, CA, USA; Fig. S1B). A custom-made 3D-printed extension with a hole on top to fit the prey-item holder was attached to the force transducer to level its tip with the surrounding surface. The forces of 5 individuals (n = 3–11, weight = 0.086 g ± 0.014 g) were measured on three consecutive days, with at least three strikes per animal per day, resulting in a total of 24 recorded strikes from all animals combined. Small house crickets (*Acheta domesticus* (linnaeus, 1758)) of three different size categories (0.3–0.4 cm; 0.5–0.7 cm; 0.8–1.1 cm) were used for the experiments. The house crickets were cooled down, their size measured, and they were subsequently attached to the prey item holder using beeswax and fitted into the experimental setup. Before the experiment, mantises were weighed using an AG204 DeltaRange high-precision weighing machine (Mettler-Toledo, Columbus, Ohio, USA), and values were saved in an Excel sheet. The mantis was placed at the base of a platform, from which it approached the prey item. After the animal successfully struck the prey item attached to the force transducer, the measured force–time curves were saved with the program AcqKnowledge 3.7.0 (BIOPAC Systems, Inc., Goleta, CA, USA) as a “.*acq*” file. The peak forces and median values of each strike were extracted and transferred into an Excel sheet. Subsequently, the data were statistically evaluated.

### Confocal laser scanning microscopy (CLSM)

An adult specimen of *H. orlovi* was cooled and euthanized using CO2, killed, and dissected before the measurements. An individual trochanter was dissected and placed on an object slide embedded in glycerine (99.9%) and covered with a high-precision coverslip. For the CLSM investigations, a Zeiss LSM 700 (Carl Zeiss Microscopy, Oberkochen, Germany), with the following laser lines (wavelengths of 405, 488, 555, and 639 nm) and emission filters (BP420–480, LP490, LP560, LP640 nm) were used (e.g. Figure 1 in Büsse and Gorb [65]) to determine the material composition of specific areas of the insect cuticle by depicting its autofluorescence (cf. Figure 1 in Büsse and Gorb [65]). The resulting data were processed to generate maximum intensity projections (*mip*) using ZEN2008 software (Carl Zeiss Microscopy, Oberkochen, Germany). The *mip* visualizes the combined autofluorescence signals in every pixel, allowing for an estimation of the cuticle components as well as their material composition [66–68].

For further information on the background of this analysis and the determination of the cuticle properties based on its autofluorescence signal, please refer to Michels and Gorb [67]. The color code of the autofluorescence for the interpretation of the images (Fig. 3F and G) is as follows: red—sclerotized and rather stiff cuticle; a combination of blue, green, and red – medium stiff and more flexible cuticle in comparison to the first one; blue – highly flexible cuticle, characteristic of the rubber-like protein resilin [69]. It is important to note that although these results allow for a qualitative assessment, they do not represent a quantitative measurement of cuticle properties. Images were subsequently post-processed using Affinity Designer 2 (Serif (Europe) Ltd., Nottingham, United Kingdom).

### 3D-Printing

The 3D-printed proof-of-concept model (Fig. 3A) was designed using the CAD software Autodesk Fusion 360 (Autodesk, San Francisco, California, USA), and subsequently manufactured using a Prusa MK3.5S (Prusa Research, Prague, Czech Republic), with a 0.4 mm nozzle and Extrudr PLA NX2 MATT filament (Extrudr | FD3D GmbH, Lustenau, Austria).

### Mechanical testing

The model for mechanical testing (Fig. 3A) includes two different artificially made trochanters (*atr*): one completely non-deformable (*atrH*, Fig. 3B) and one with a deformable structure (*atrS*, Fig. 3C). The deformability of the trochanter is achieved through a deformable double-spiral generated by using the Double-spiral design software [51], with the following parameters: Archimedean spirals; $\theta_{0} = 0 \times \pi \left( {rad} \right)$ and $\theta_{max} = 2 \times \pi \left( {rad} \right)$; spiral 1 with $k = 2$ and $r_{0} = 11$; spiral 2 with $k = 2$ and $r_{0} = 14.5$; parameters for spiral 3 and 4 are derived from spiral 1 and 2. Both *atr* feature attachment points (*ta*) for a threat (*th*) and an axis of rotation (*ra*). The spatial orientation of the axis, the muscle insertion point (attachment point of the threat) and its running direction are based on our knowledge from *µCT* data analysis. For the testing, the *atr* was fitted on a baseplate (*bp*), with a bearing at the rotational connection of the *atr* to the testing set-up, to allow for smooth rotation. Furthermore, we included a locking structure on the *bp* that resembles the connection between the grooved trochanter and the clasp-like shape of the enlarged coxal outgrowths (coxal lobes; Fig. 3A, *ls*). The locking structure represents the antagonistic force of the contracting flexor muscle as well as the biological locking structure, keeping the coxa and femur locked in position by generating friction forces. The baseplate is fixed on a base structure (*bs*). In the experiments, we tested the influence of an energy-storing structure within the trochanter against a case without this kind of structure. In our experimental series, both types of *atr* were put into the setup individually and tested using a ZwickiLine uniaxial testing machine (Zwick Roell, Ulm, Germany), equipped with a 500 N load cell (Xforce P load cell, Zwick Roell). The *ta* was connected to the testing machine using a non-deformable threat (*th*). At the beginning of the experiment, the tip of the trochanter was gently pressed into the *ls* until fully locked (Fig. 3D). Subsequently, tensional load was applied to the threat at a constant rate of 10 mms^−1^ (Fig. 3E) until the *atr* came loose from the locking structure and swung free (Fig. 3F).

In a second experimental approach, a differing *bp* was used in the setup, with a slightly larger gap in the locking structure that generated much less friction. All tests were filmed at 1000 frames per second. Exemplary videos can be seen here: *atrH* in high friction scenario (Movie S3), *atrS* in high friction scenario (Movie S4), *atrS* in low friction scenario (Movie S5). All trochanters had a dark marking (*ts*) at the same position to allow for subsequent motion-tracking analysis (Fig. 3D–F) to calculate the velocity after losing connection to the locking structure. In this manner, eight artificial trochanters were manufactured and tested in the two setups (four deformable, four non-deformable) and tested 6–8 times each for both experimental approaches. In the resulting videos, the dark spot on the trochanter was tracked using the visual effects software Adobe after Effects CS6 (Adobe Systems Software, San José, CA, USA) to obtain frame-by-frame information using the software’s “Motion Tracking” workspace and its tracking algorithm. The X and Y coordinates of each tracker were extracted as a “.*txt*” file for subsequent processing and statistical analysis, using a JavaScript plugin from Koehnsen et al. [64]. Using R, the linear velocity of the point was calculated. The values for obtained forces and calculated velocities can be found in Table S3. Subsequently, the obtained data were evaluated using a linear mixed-effects model (*LME*), to obtain information about maximum forces ($\log \left( {force} \right)\sim type \times friction + \left( {1\left| {sample} \right.} \right)$) and maximum velocities ($velocity\sim type \times friction + \left( {1\left| {sample} \right.} \right)$). Force represents the measured maximum forces; velocity represents the calculated maximum velocities. For the analyses, force and velocity were treated as the response variables. Because the force data exhibited heteroscedastic residuals on the original scale, data were log-transformed prior to analysis. Type represents the two types of *atr* (*atrH* and *atrS*); friction represents the high and low friction scenarios. Type and friction were treated as fixed effects and analyzed for both main and interaction effect. The sample represents the different samples and was included as a random intercept in both models. Fixed effects (type and friction) were evaluated using Type III analysis of variance; effects within type (*atrH* and *atrS*) were subsequently quantified using estimated marginal means and pairwise contrast analysis. All relevant outputs from the *LME*s can be found in Tables S6–S9.

### Statistical analysis

The statistical computing programs R 4.0.0 (The R Foundation for Statistical Computing, Vienna, Austria) and RStudio 1.2.5033 “Orange Blossom” (RStudio PBC, Bosten, Massachusetts, USA) were used to further analyze the obtained data and generate plots with the R package “ggplot2”. Results of all tests were treated as follows: p = α < 0.05 ≙ significant.

## Results and discussion

### General characteristics of the raptorial forelegs and the predatory strike

The habitus of the sub-chelate-type raptorial forelegs of *H. orlovi* (Fig. 1A) shows remarkable similarities to those found in representatives of Thespidae [36, 42, 70]. As is typical for mantises, the proximal end of the first leg segment (coxa) shows two articulatory points with the first body segment (prothorax; one with the pleural process and one with the trochantine that is flexibly connected to the katepisternum (cf. Figure 2 in Bäumler et al. [29])) It allows movements of the coxa in all directions: even rotations—resulting in an extreme movability and versatility of the basal leg segment [71], and thus, the entire leg. In *H. orlovi*, the range of motion is more restricted due to the specific shape of the lateral body wall (e.g., lateral lobes of the prothorax). Scanning electron microscopy (SEM) analysis gives more detailed insights into distinctive external morphological features (Fig. 1C–E). Noteworthy is the enlarged and reinforced crest on the first leg segment (posterior coxal ridge (*cxr*, Fig. 1C)), which runs centrally from the proximal to the distal end on the posterior surface, becoming smaller in the distal direction, terminating in one of the articulation points of the first and second leg segments (posterior articulation point between coxa and trochanter). Enlarged outgrowths (anterior and posterior coxal lobes (*cl*, Fig. 1D, E) protrude from the distal end of the coxa (Fig. 1C–E), overtopping part of the trochanter. The shape of the trochanter shows corresponding groove-like indentations (on anterior and posterior surfaces, respectively), fitting the shape of both enlarged outgrowths of the first leg segment (*cl*, Fig. S3). Furthermore, the trochanter is noticeably narrowed in the most proximal part and shows folded surface areas close to the joint (Fig. S3). An interactive 3D model of the trochanter can be found following this link: https://skfb.ly/pAOwA. As shown in a comprehensive overview by Oufiero [72], mantodeans foreleg article segments exhibit great diversity in size, proportions and shape. In contrast to other mantodeans, the third leg segment (femur) of *H. orlovi* is dorso-ventrally thinned, especially at the distal end, and bent in a wave-like shape along its longitudinal axis (Fig. 1 A, C). The tibia of *H. orlovi* is particularly interesting: it is proportionally shortened in comparison to the “standard” mantis morphology [72] and shows a reduction and different arrangement of spines, as already observed by Brannoch et al. [30]. The four most distal spines, especially the most distal spine (apical tibial spur (*ats*)), are significantly enlarged and bent to face more distally, as was also observed for *Thesprotiella simpira* (Fig. 3A; cf. Figure 4 in Rivera and Callohuari [27]). Furthermore, the spines themselves are less curved than usually observed in other mantises (see, for example, the *ats*, Fig. 1A, C). This results in a straighter shape that probably eases penetration of prey items, but could also lead to an increased risk of releasing the prey again, and simultaneously represents a mechanism for stress reduction in this biomechanically challenging movement [27].

The praying mantis species used in this study (*H. orlovi*) exhibits a distinctively altered predatory behavior compared to most other mantises: grasping vs impaling. The general predatory strike can be divided into a longer approaching phase, where the mantis gets into an ideal striking position, followed by a significantly shorter sweeping phase, which ends with the prey item being captured [40]. The majority of mantodeans fully extend their forelegs in the sweeping phase, to bring the distal part of the leg (tip of the tibia) behind the prey item, subsequently closing the femur-tibia joint and trapping the prey in a grasping-like motion [31, 34, 38–40, 73]. In contrast, *H. orlovi* uses its most distal tibial spine (*ats*) to impale its prey item to prevent an escape. The results of our high-speed video analysis have revealed the movement pattern, which is consistent with that observed by Rivera and Callohuari [27] for a representative of the Thespidae. A step-by-step visual documentation of the process in *H. orlovi* can be seen in Fig. 2C–E. When the prey item is detected, the mantis focuses its attention on it, positions itself over the prey and performs the following steps: (a) preparing the leg to strike (closing the coxa-trochanter and femur-tibia joints while the body-coxa joint remains open; Fig. 2C), (b) approaching the prey item (opening the femur-tibia joint, simultaneously tilting its head backwards until the femur-tibia joint reaches an almost perpendicular orientation; Fig. 2D), (c) sweeping phase (the body-coxa and femur-tibia joints do not change opening position, the coxa-trochanter joint opens in a rapid motion, catapulting the tip of the tibia towards the prey item to impale it; Fig. 2E), (d) prey capture (the motion stops, when the *ats* cannot further impale the prey or gets into contact with a surface or structure that stops it). A short *HSV* of an exemplary predatory strike, filmed at 10,000 frames per second, can be seen in Movie S1. Our *HSV* analysis revealed that the sweeping phase in *H. orlovi* is significantly faster compared to the commonly found grasping strategy in other mantises. While the general sweep of other mantises lasts ~ 0.01–0.03 s [38–40], the impaling sweeping motion of *H. orlovi* is extremely fast, with ~ 0.0016 s for the sweeping motion (from the closed coxa-trochanter joint until the impaling of the prey item). Due to the extreme velocity and resulting forces, high loads are transferred onto the long femur when the *ats* hits the prey item. Its thinned, prebend shape likely results in increased flexibility in the dorsal direction, which could function as a dampening mechanism to prevent structural failure. Although the predatory strike in mantodeans was previously described to be directly muscle-driven [41], the observed rapid impaling motion exceeds the limits of muscle-driven movements, prompting the calculation of mass-specific *mpo*.

### Force measurements

To investigate this phenomenon, the force of the predatory strike of *H. orlovi* was measured, as described in the Materials and Methods section, by recording strikes on different days using three differently sized prey items. Although the resulting peak striking forces differed significantly between the two specimens, when calculating the body-mass-specific forces, these differences disappeared. The size of the prey item had no significant effect (Fig. S4). Animals generated a mean force of *F* = 17.62 mN ± 6.41 mN (Fig. S4). All measurements can be found in Tables S4 and S5 and were used, together with the kinematics from the *HSV* analysis, to calculate the mechanical power output of the strike in two different approaches, described in the supplementary Methods S1. The calculated muscle mass-specific *mpo* was 58,839.96 Wkg^−1^ ± 22,074.86 Wkg^−1^ for the first approach, and 30,678.94 Wkg^−1^ ± 4306.61 Wkg^−1^ for the second approach. Although the resulting values from both methods vary widely, they still exceed the reported limits of direct muscle performance of 100–500 Wkg^−1^ by far [2–5, 74–78]. In comparison, e.g. trap-jaw ants reach mass-specific power outputs of ~ 21,000 Wkg^−1^ [79], planthoppers reach ~ 28,000 Wkg^−1^ [80]. We therefore assume that a power amplification mechanism must be in place to drive the observed process.

### Musculature

Our results from morphological examination using micro-computed tomography (*µCT*) and dissection revealed that the muscular system is surprisingly similar to that of other mantises, with attachment points remaining comparably consistent. We confirmed 14 extrinsic and 14 intrinsic muscles associated with leg movements for *H. orlovi* (only the small muscle I*scm6* is missing in comparison to other mantodeans [29]). A complete list and comprehensive overview of the attachment areas is presented in Table S2. For a 3D-visualization and an interactive 3D model of the entire musculature set-up of the leg movement-associated musculature, please refer to Figs. S5 and S6 as well as the following links: https://skfb.ly/pAPwX, https://skfb.ly/pAPwY and https://skfb.ly/pAPwZ. In our further analysis, we focused on the proposed ‘catapult’ mechanism and identified seven muscles to be involved in its opening and closing (Fig. 2A). Functionally, and based on their attachment sites, these muscles can be divided into five groups, and to facilitate all further descriptions, they will be named as follows: extensor muscle 1 (running from the prothorax to the proximal trochanter; *Idvm19* and *Ipcm8*), extensor muscle 2 (running from the proximal coxal surface to the proximal trochanter; *Ictm4* and *Ictm5*), flexor muscle (running from the proximal coxal wall to the middle of the trochanter; *Ictm1*), locking muscle 1 (running from the distal coxal surface to the proximal trochanter; *Ictm6*) and locking muscle 2 (running from the distal coxal surface to the middle of the trochanter; *Ictm3*) (Fig. 2A). Interestingly, taking the extensor muscles altogether, they appear to occupy a comparably larger portion of the first leg segment (coxa), which is not as pronounced in other species. While these antagonistic groups of muscles are also used during locomotion, they are likely co-activated in the context of the catapult mechanism [28, 41].

### Proposed power amplification mechanism

The proposed power amplification mechanism is mainly based on elastic energy storage in combination with a latch-like locking mechanism. This mechanism is displayed simplified in Fig. 1B and in more detail in Fig. 2H, I, L and M, showing the associated groups of muscles as well as the pivot point and area of deformation in the second leg segment (trochanter). In Fig. 2H, the foreleg with the corresponding muscles is shown in a relaxed state. During the approaching phase, the flexor muscle and the locking muscle 1 contract, bringing the first and third leg segments (coxa and femur) into very close contact, as their profiles fit well together (Fig. 2C, I). The matching profiles of the coxa and femur support a secure positioning during the preloading process of the *LaMSA* system. Additionally, both enlarged coxal outgrowths (anterior and posterior coxal lobes) of the distal coxa (Fig. 1D, E) come into close contact with the corresponding grooves of the trochanter. Here, the locking muscles 1 and 2 are likely co-activated, to pull the grooved trochanter closer into the clasp-like shape of the two enlarged coxal outgrowths, creating a locking mechanism (Fig. 2D, F, L). When the leg (i.e., the femur) is locked in place by the contracted flexor muscle and locking muscles, the large extensor muscles 1 and 2 start to contract, applying load to the proximal part of the trochanter (Fig. 2L). Now, a key morphological feature comes into play—the modified and deformable trochanter (Fig. S3). Due to the applied force, the proximal part of the trochanter gets deformed, bending dorsally, acting as an energy storage device (Fig. 2L). The *HSV* footage further supports this hypothesis, as a significant deformation of the trochanter is visible in this phase of the strike (Fig. 2F, G). Shortly before the sweeping phase, the joint between the third and fourth leg segment (femur-tibia joint) is opened nearly 90° (tibia is now perpendicular to the femur) (Fig. 2D, F, L). To initiate the sweeping motion, the flexor muscle and the locking muscle 2 relax, releasing the grooved trochanter from its clasp, giving way to opening the joint of the first and second leg segment (coxa-trochanter joint) (Fig. 2E, J, M). The stored energy of the deformed trochanter unloads, adding up to the extensor muscle energy, resulting in a highly accelerated movement (Fig. 2E, J, K, M). The significant deformation of the trochanter becomes even more pronounced when comparing it to its relaxed state after the strike (Fig. 2J, K). This deformation is facilitated by both (1) its proximally thinned and creased shape (Fig. S3) and (2) its material composition, as revealed by confocal laser scanning microscopy (*CLSM*; Fig. 2B). The joint appears bright red, indicating high sclerotization, securing stability and a proper movement and force transmission (Fig. 2B). The proximal part of the joint reveals an orange-colored ventral area, indicating a semi-sclerotized cuticle, and a bright red area, indicating a strongly sclerotized cuticle, that closely resembles the saddle-shaped cuticle of mantis shrimps [23]. In this context, it has been shown that a specially shaped sclerotized cuticle can be employed as a region for energy storage to increase the velocity of an arthropod’s motion [23]. This material composition and distribution support a strong connection of the joint cuticles while simultaneously enabling deformation of the proximal part of the trochanter, where all extensor muscles insert. The evolution of this specialized predatory mechanism raises further intriguing questions about the selective pressures and environmental factors that drove its development. While the impaling hunting strategy of *H. orlovi* enables extremely fast strikes, it likely involves functional trade-offs, potentially sacrificing versatility, influencing the range of potential prey items. This possible trade-off exemplifies the complex interplay between morphological adaptations and ecological specialization in the evolution of predatory strategies. Due to a similarity in habitat preference (i.e., on mosses in humid forest environments), and as the observed predatory behavior is remarkably similar in both species, it is likely that *H. orlovi* and the previously investigated *T. simpira* not only share morphological traits, but also face a similar availability in prey items in their microhabitat. Therefore, they face comparable ecological challenges and are under similar selective pressure [27, 81–84]. This habitat is mainly characterized by higher humidity, a rather complex structure and is dominated by minute soft-bodied arthropods (~ 0.35 mm body length and potentially smaller) [85–88]. It was shown that while *T. simpira* is able to prey on larger insects (i.e., larger domestic flies), it actively focuses on minute arthropods, which are impaled with high precision [27]. A similar trend is noticeable for *H. orlovi*: at least in captivity—where even larger nymphs were observed hunting spring tails and other comparably small-bodied arthropods on a regular basis. When larger flies (i.e., gold flies) were introduced to the terrariums, adults took longer durations to recognize and impale them, in comparison to, e.g., the much smaller fruit flies (*Drosophila hydei*). Rivera and Callohuari [27] argued that by actively keeping a focus on comparably smaller arthropods during foraging, instead of expanding the favored prey size as is typically observed for predators during growth, the animals make best use of the available prey of their chosen microhabitat.

### 3D-printed model

A key morphological feature of the described *LaMSA* system is the modified trochanter, which becomes deformed during movement, acting as an energy storage unit. While elastic energy storage through cuticle deformation is a well-established principle in biological power-amplification systems [57], the specific combination of cuticle deformation, frictional interlocking, and passive release proposed here still represents a mechanically non-trivial interaction. To test our hypothesis of the interplay of the described components of the *LaMSA*, and present it in an informative and tangible way, we developed a simplified bio-inspired 3D-printed proof-of-concept model. Our model is based on our comprehensive morphological and experimental analysis, including the main aspects of the proposed *LaMSA* system in the design (e.g., the attachment areas of muscles and the relative placement of the rotational axis; Fig. 3A). Importantly, the model is not intended to quantitatively replicate biological performance, but to test whether the proposed geometry and interaction of elastic deformation and interlocking are, in principle, sufficient to generate a power amplification, as was observed in the animals. The experiments were conducted as described in the Materials and Methods section, and all results can be found in Table S3. A boxplot, containing the results of the experiments and the subsequent linear mixed-effects model (*LME*) analysis can be seen in Fig. 3G (resulting forces) and H (calculated maximum angular velocities). All relevant outputs of the *LME* can be seen in Tables S6–S9. The *LME* revealed that the friction scenario significantly affects the resulting forces (Table S7), with the low friction scenario (*L*) having a negative influence on the resulting forces for the two *atr* types (Table S8). Overall, this effect of friction on the forces does not differ between both *atr* types (Table S7). Unlike force, velocity differs between the *atr* types when averaged across the friction scenarios (Table S7), but the effect of the friction scenarios differs significantly between both *atr* types (Table S7). The Type III analysis of variance confirmed a strong interaction of the fixed effects, with friction affecting velocity in a type-specific manner (Table S7). While the calculated velocities show no differences between the friction scenarios for the *atrH*, they differ significantly between the friction scenarios for the *atrS* (Table S8). For the *atrS*, significantly higher velocities were measured in the high-friction compared to the low-friction scenario (Table S3). Therefore, having a locking structure (*ls*; in the biological case, the interplay of the flexor muscles and the locking structure) that works antagonistically to the pulling of the threat (in the biological case, the extensor muscles), significantly increased the obtained forces, as well as the resulting maximum velocities of the *atrS* (in the biological case, the opening of the trochanter-femur joint).

The *LME* further revealed that individual samples (*N* = 4 per *atr* type) contribute strongly to the variability in measured forces and velocities (Table S9), which does not come as a surprise. This is presumably due to the tolerances in the manufacturing process of the 3D-printed *atr*. Even minor deviations in thickness or the structure can have a big influence on the friction between the *atr* and the *ls*. Rather than representing a shortcoming of the model, this sensitivity reflects an intrinsic property of contact-based power-amplification systems, in which small geometric differences can strongly affect frictional behavior and release dynamics. Generally, in the low friction scenario, both *atr* showed comparably low and rather similar forces (*atrH* mean = 0.55 N; *atrS* mean = 0.39 N) and maximum velocities (*atrH* mean = 0.072 ms^−2^; *atrS* mean = 0.056 ms^−2^; Fig. 3G, H; Table S3). In the high friction scenario, the measured forces for both *atr* are, again, similar and comparably higher than in the low friction scenario (*atrH* mean = 22.38 N; *atrS* mean = 18.63 N; Fig. 3G, H). Interestingly, the calculated maximum velocities for the high friction scenario are similar for the *atrH* (*atrH* mean = 0.078 ms^−2^) compared to the low friction scenario, but significantly higher for the *atrS* (*atrS* mean = 0.205 ms^−2^; Fig. 3G, H; Table S3). When comparing the measured tensile forces with the resulting maximum velocities, higher forces seem to result in higher velocities, up to a certain threshold. The threshold is presumably based on the point where the elastic element (double spiral) reaches its maximum deformation. From there, additional tensile energy cannot be stored in the spiral by further deformation, and therefore, the resulting maximum velocity does not increase any further. This behavior is consistent with a mechanically limited spring element, reinforcing the interpretation of the system as a passively activated *LaMSA* mechanism. As an example, while *atrS2* shows the highest forces in the high friction scenario (Fig. 3G), the corresponding maximum velocities are similar to those in most other *atrS* (Fig. 3H). Nevertheless, in contrast to the *atrH*, a correlation between the applied tensile forces and the resulting maximum velocities is present in the *atrS*. This correlation is particularly noticeable in the high-friction scenario of the *atr3*: the measured forces (Fig. 3G) and resulting maximum angular velocities (Fig. 3H) are much smaller than in the other samples of the *atrS*. While the calculated maximum velocity values are slightly higher than the ones obtained for all of the *atrH*, the deformable *atrS3* required much less force to achieve it. When considering the forces, it becomes apparent that the *atrS3* must generate less friction in comparison to the remaining *atrS*. This is further supported by the video footage (exemplary video can be seen in Movie S5): here it is clearly visible that, in comparison to the other *atrS*, the spiral does not reach its maximum possible deformation before the *atrS* is released from the *ls*. Overall, our results have shown an increased velocity of the system, including the deformable trochanter (*atrS*). These findings demonstrate that elastic deformation combined with frictional interlocking is sufficient to produce power amplification in a purely mechanical system. For the biological system, the deformable part of the trochanter in combination with the locking structure between the trochanter and femur, very likely results in the observed *mpo* and maximum velocity generation during the predatory strike.

Using this simplified model, we have shown the advantage of the presence of a *LaMSA* in this system. We have shown that the geometric arrangement and frictional contact can together ensure the energy storage process and trigger the rapid release, without active control or specialized materials. Furthermore, introducing a deformable elastic element increased the achievable velocity relative to a rigid analogue under otherwise comparable loading conditions. The conclusions drawn from the physical model are restricted to qualitative demonstration of the simplified mechanism and do not allow for direct conclusions of absolute biological performance, scaling relationships, or energetic efficiency. Our results further highlight the possible dual functionality of this biological structure. Under smaller load (Fig. 3G, H), with no deformation of the double spiral, both versions of the trochanter behave as rigid bodies. In the animal, this means that the leg can be used for walking and other movements, without always preloading the *LaMSA* system. Under higher loads, the elastic element deforms significantly, releasing its stored energy in the subsequent motion, resulting in an increased velocity in contrast to the rigid trochanter. In our model, as well as in the investigated organism, the deformable structure works as a mechanical relay. It allows the system to generate higher kinetic energy in a shorter time than would be physiologically possible by direct muscle contraction. While the specific double-spiral geometry represents only one possible realization of the elastic element, it serves as a convenient and controllable abstraction of the biological deformation component. By adjusting the load-bearing capacity of the elastic element (in the artificial system—double-spiral), the behavior of the system could further be altered, as was shown in previous studies [51], to even better mimic the behavior in the animal. The double spiral could, for example, be manufactured to withstand higher loads before starting to deform, to achieve a better differentiation between the two behaviors of the dual functionality. This could be achieved by, e.g., changing the extrusion height of the spiral, but also by adjusting the parameters in the Double-spiral design software [51]. For example, decreasing the angle of rotation ($\theta$) would decrease the number of coils in the double-spiral, therefore reducing the deformability. Increasing $\theta$ would increase the number of coils and the spiral’s deformability but lead to a larger area the the structure accommodates. The scaling is another important factor, as with increasing size, the thickness of the spiral increases, reducing its flexibility. Changing the material of the double-spiral exclusively could alter the behavior of the system, potentially increasing its performance. Yet one of the advantages of the presented model lies in the manufacturing process, as it is produced in one coherent piece. Through geometry alone, deformability is created, eliminating the need to manufacture and assemble multiple parts. Depending on the requirements of the set-up, the double-spiral is meant to be included in (e.g., size of the spiral, how much force it needs to withstand without deformation), a distinct combination of designing and manufacturing parameters is needed. Future work may further explore alternative geometries and general material choices to systematically examine the parameters of this mechanism. Finite element analysis could be employed to systematically investigate stress distributions, deformation patterns, and energy storage capacity of the elastic element under varying loading conditions. This would enable a more quantitative assessment of design parameters and boundary conditions—e.g., how geometric parameters, material properties, and scaling influence energy storage and release dynamics—complementing the experimental physical proof-of-concept presented here.

## Conclusions

Through comprehensive morphological and experimental analysis, using micro-computed tomography, scanning electron microscopy, confocal laser scanning microscopy, high-speed videography and force measurements, we uncovered a set of significant morphological adaptations at the level of the exoskeleton, cuticle composition and muscular setup that enable the exceptional impaling hunting strategy of *H. orlovi*. In contrast to most other mantodeans, which hunt with a grasping-like motion, *H. orlovi* uses its spear-like modified raptorial forelegs to impale its prey. The latch-mediated spring actuation system, observed in this species, represents a remarkable example of evolutionary adaptation for predation. The described mechanism enables the animals to store and rapidly release energy, resulting in an exceptionally fast impaling motion used to capture prey, with a mass-specific power output of ~ 30,000 Wkg^−1^. The key morphological feature of the system lies in its ability to accumulate potential energy over a relatively extended period of time and then release it rapidly, generating a powerful strike that far exceeds the mass-specific power output possible through direct muscle action alone. This deformation of the trochanter is facilitated by both its shape and material composition, as is revealed by confocal laser scanning microscopy analysis. The animals exhibit an ideal combination of morphological and behavioral traits, linked to their complex, structured habitat, filled with minute-sized arthropods. Their relatively small body size helps to navigate the environment while staying safe and camouflaged. Additionally, the impaling hunting strategy represents a comparably compact movement for hunting, ideal when prey is potentially located in places unfeasible for predation using a grasping movement (e.g., crevices). The impaling hunting mechanism represents a comparably compact and simple design of a biological power amplification system. Our 3D-printed proof-of-concept physical model and the associated tests have shown that while the measured tensile forces of both artificial trochanters are similarly affected by the two different friction scenarios, the calculated maximum velocity shows a type-specific friction response. Despite the fact that technical power amplification systems are subject to different scaling constraints, the underlying morphological and mechanical principles may nevertheless provide conceptual guidance for bio-inspired engineering design strategies, highlighting the broader implications of such studies in fields beyond evolutionary biology and biomechanics. Future studies could employ finite element analyses to further elucidate stress distributions and deformation dynamics of the elastic element, enabling a more quantitative exploration of the system’s functionality. This study not only expands our understanding of the diversity and evolution of predatory adaptations in mantises but also demonstrates the value of integrating morphological, kinematic, and biomechanical analyses in uncovering novel biological mechanisms. It further demonstrates the potential for seemingly minor morphological modifications to drive significant functional and ecological shifts, potentially opening new ecological niches.

## Supplementary Information

Additional file1 (DOCX 6294 kb)

Additional file2 (MP4 19271 kb)

Additional file3 (MP4 73796 kb)

Additional file4 (MP4 20996 kb)

Additional file5 (MP4 20869 kb)

Additional file6 (MP4 21115 kb)

Additional file7 (MP4 21988 kb)

## Data Availability

All data supporting our findings are presented in the paper and supporting information, respectively. The µCT dataset used and analyzed during the current study is available here: 10.5281/zenodo.18377868.
