# Supplementary material for: A striking difference: biomechanics of the impaling hunting strategy of a moss mantis
Source: Front Zool. 2026 Apr 11;23:15. doi: 10.1186/s12983-026-00610-9 (PMC13094072; doi:10.1186/s12983-026-00610-9)
Supplement: Supplementary file 1 — Additional file1 (DOCX 6294 kb) [file 12983_2026_610_MOESM1_ESM.docx]

**Supplementary materials for**

A striking difference: Biomechanics of the impaling hunting strategy of a moss mantis

Fabian Bäumler^1^*, Stanislav N. Gorb^1^ and Sebastian Büsse^2^

^1^ Functional Morphology and Biomechanics, Institute of Zoology, Kiel University, Kiel, Germany

^2^ Cytology and Evolutionary Biology, Institute of Zoology and Museum, University of Greifswald, Greifswald, Germany

*Corresponding author

**Email**: fbaeumler@zoologie.uni-kiel.de

**This document includes:**

Supplementary Methods S1

Supplementary Figures S1-S6

Supplementary Tables S1-S6

Legends for supplementary Movies S1 to S6

Movies S1 to S6 are provided separately

**Methods S1**

Mass-specific power output

To allow for an estimation, whether the observed predatory strike is a purely muscle driven movement, or employs power amplification, the mass-specific power output was calculated using the following equation

$$\boldsymbol{p=}\frac{\boldsymbol{P}}{\boldsymbol{m}_{\boldsymbol{mus}}}$$

where $\boldsymbol{p}$ [Wkg^-1^] is the mass-specific power output, $\boldsymbol{P}$ [W] the mechanical power output, and $\boldsymbol{m}_{\boldsymbol{mus}}$ [kg] the mass of the actuating musculature in the coxa.

The $\boldsymbol{m}_{\boldsymbol{mus}}$ was calculated using information from micro-computed tomography (*µCT*) analysis. From the *µCT* scan, the volume of the musculature associated with the opening of the coxa-trochanter joint was obtained. The volume was then multiplied with literature data (1060 kgm^-3^) that is commonly used for insect muscle density (1). During the preparation process for the *µCT* scan (i.e., Bouin solution fixation, ascending alcohol series, critical point drying), the muscle tissue may have exhibited shrinking artefacts to an unknown extent. The extent of this phenomenon strongly depends on the used chemicals as well as the duration of the treatments, causing losses from 5% to over 50% of the original tissue volume (2–5). Concerning our protocol, Bouin solution is generally reported to cause only small shrinkage artefacts (4), and the exposure time of our specimen to chemicals was very low. Nevertheless, to account for any possible shrinkage artefacts and the thereby caused lower determined muscle volume, we performed all calculations that involve the muscle mass based on *µCT* volume, using two different volumes: the original volume obtained from the *µCT* scan, and the same volume increased by 25%.

To calculate values for the mechanical power output $\boldsymbol{P}$, we used in two different approaches.

First approach

In the first approach, we used the equation

$$\boldsymbol{P=}\frac{\boldsymbol{W}}{\bar{\boldsymbol{t}}}$$

where $\boldsymbol{W}$ [J] is the work done by the musculature associated with the opening of the coxa-trochanter joint, and $\bar{\boldsymbol{t}}$ [s] the average duration of the opening of said joint. To calculate values for the work $\boldsymbol{W}$, the equation

$$\boldsymbol{W=\tau\times}\bar{\boldsymbol{rad}}$$

was used, where $\boldsymbol{\tau}$ [Nm] is the torque and $\bar{\boldsymbol{rad}}$ is the average radian. The average duration $\bar{\boldsymbol{t}}$ and average radian $\bar{\boldsymbol{rad}}$, which were overcome during the opening of the coxa-trochanter joint, were obtained from the previously described high-speed video (*HSV*) and motion tracking analysis. Here, the average value of all analysed videos was taken as a single value for subsequent power output calculations. Lastly, the torque $\boldsymbol{\tau}$ was calculated using the equation

$$\boldsymbol{\tau= F \times r}$$

where $\boldsymbol{F}$ [N] is the force and $\boldsymbol{r}$ [m] is the radius.

The radius $\boldsymbol{r}$ was measured using the *µCT* data with Amira®s 3D measurement tools, as the distance between the pivot point and the point of muscle attachment. Here, the distance between the two pivot points (a) and the distance between the point of muscle attachment and both pivot points respectively (b and c) were measured 15 times. The mean of the values was taken to construct a triangle. Subsequently, a line was constructed from the middle of site a to the point of muscle attachment and taken as the radius $\boldsymbol{r}$. The information from the force measurement experiments was used as the force value $\boldsymbol{F}$ for the equation.

Second approach

In the second approach, we used the equation

$$\boldsymbol{P= \tau\times}\bar{\boldsymbol{\omega}}$$

where $\boldsymbol{P}$ [W] is the mechanical power output, $\boldsymbol{\tau}$ [Nm] is the torque and $\bar{\boldsymbol{\omega}}$ [ms^-1^] is the average angular velocity. The average angular velocity $\bar{\boldsymbol{\omega}}$ was obtained from the previously described high-speed video (*HSV*) and motion tracking analysis. The mean value of the average angular velocity $\bar{\boldsymbol{\omega}}$ of all investigated *HSV* was used for the calculations. The torque $\boldsymbol{\tau}$ was calculated using the following equation

$$\boldsymbol{\tau=I \times}\bar{\boldsymbol{a}}$$

where $\boldsymbol{I}$ [kgm^-2^] is the moment of inertia, and $\boldsymbol{a}$ [ms^-2^] the average angular acceleration of the opening of the coxa-trochanter joint. The average angular acceleration $\bar{\boldsymbol{a}}$ was obtained from the previously described high-speed video (*HSV*) and motion tracking analysis. The mean value of the average angular acceleration $\bar{\boldsymbol{a}}$ of all investigated *HSV* was used for the calculations. The moment of inertia $\boldsymbol{I}$ was calculated, using the equation

$$\boldsymbol{I=}\frac{\boldsymbol{1}}{\boldsymbol{3}}\boldsymbol{\times}\boldsymbol{m}_{\boldsymbol{TF}}\boldsymbol{\times}\boldsymbol{L}_{\boldsymbol{TF}}^{\boldsymbol{2}}\boldsymbol{+}\boldsymbol{m}_{\boldsymbol{TT}}\boldsymbol{\times}\boldsymbol{L}_{\boldsymbol{TF}}^{\boldsymbol{2}}\boldsymbol{+}\frac{\boldsymbol{1}}{\boldsymbol{3}}\boldsymbol{\times}\boldsymbol{m}_{\boldsymbol{TT}}\boldsymbol{\times}\boldsymbol{L}_{\boldsymbol{TT}}^{\boldsymbol{2}}$$

where $\boldsymbol{m}_{\boldsymbol{TF}}$ [kg] is the mass of the trochanter and tibia (combining musculature and cuticle), $\boldsymbol{m}_{\boldsymbol{TT}}$ [kg] is the mass of the tibia and tarsus (combining musculature and cuticle), $\boldsymbol{L}_{\boldsymbol{TF}}$ [m] is the length between the coxa-trochanter joint and the most distal part of the femur, and $\boldsymbol{L}_{\boldsymbol{TT}}$ [m] is the length of the tibia. As the tarsus is kept close to the tibia during the striking motion, its length is ignored in the calculation. The masses for $\boldsymbol{m}_{\boldsymbol{TF}}$ and $\boldsymbol{m}_{\boldsymbol{TT}}$ were calculated using information from micro-computed tomography (*µCT*) analysis, similar as described for the musculature associated with the opening of the coxa-trochanter joint. Concerning the musculature, again the original volume obtained from the *µCT* scan, and the same volume increased by 25% were used for the calculations. The masses of the cuticle parts were determined similar, by using an average value for insect cuticle density (1150 kgm^-3^) (6). To support the resulting mass values, we additionally dissected a specimen, manually weighing trochanter and femur, and tibia and tarsus, as a comparison. The results proved to be similar, with 1.06473E^-06^ kg for the calculated values (using average values for insect muscle and cuticle density) and 1.2544E^-06^ kg for the manually weighed values.


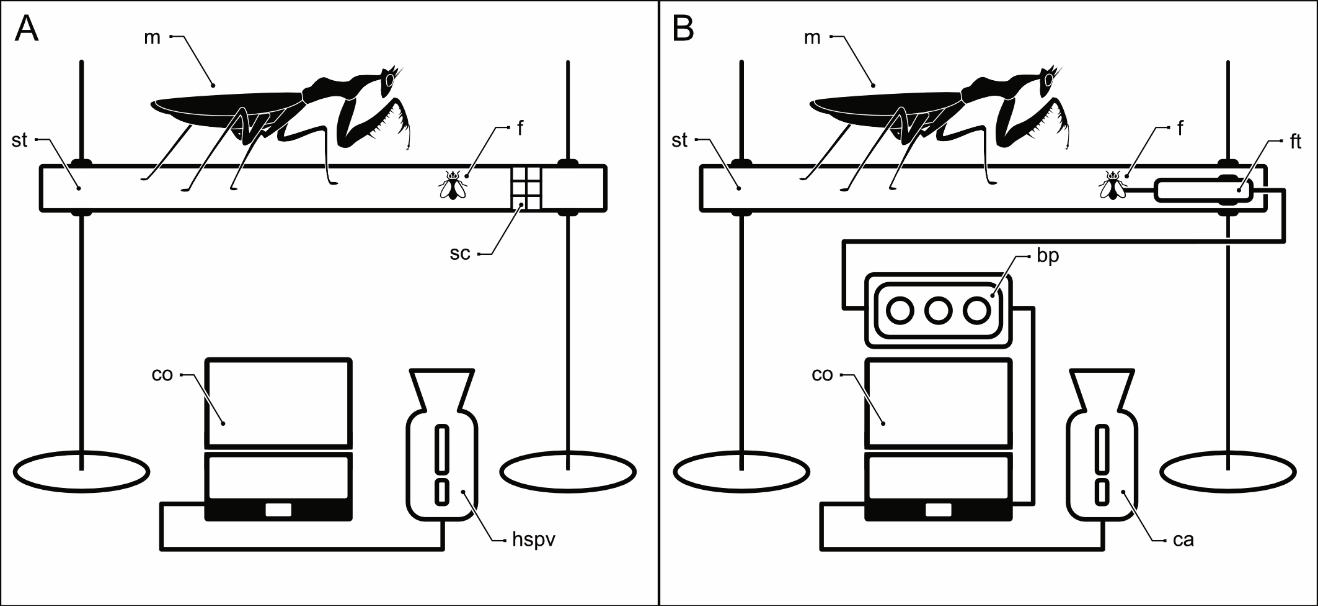
Figure S1 – Experimental setup for high-speed videography (A) and force measurements (B). Abbreviations: bp – Biopac system; ca - camera; co – computer; f – fly; ft – force transducer; hvpc – high-speed video camera; m – mantis; s – specimen; sc – scale; st – stick.


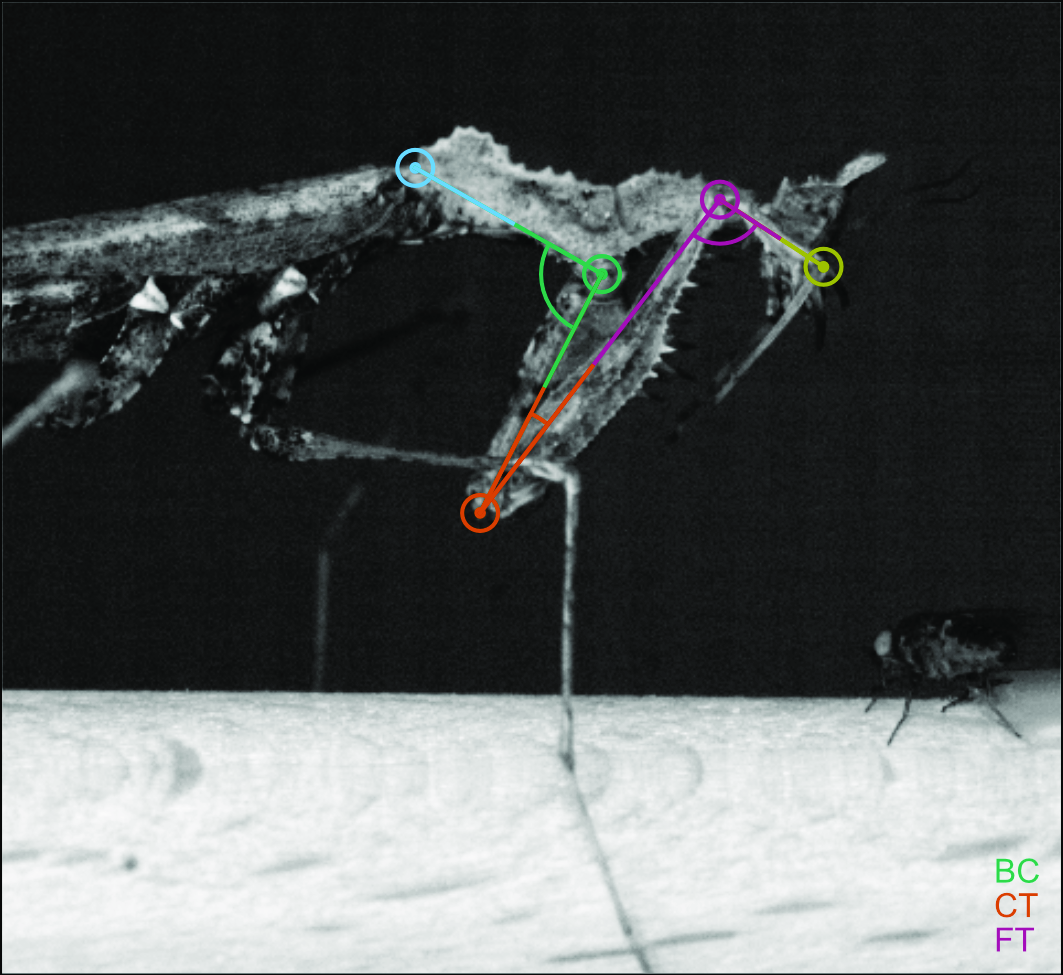
Figure S2 – Tracked points in motion tracking approach. Abbreviations: BC – body-coxa joint; CT – coxa-trochanter joint; FT – femur-tibia joint.


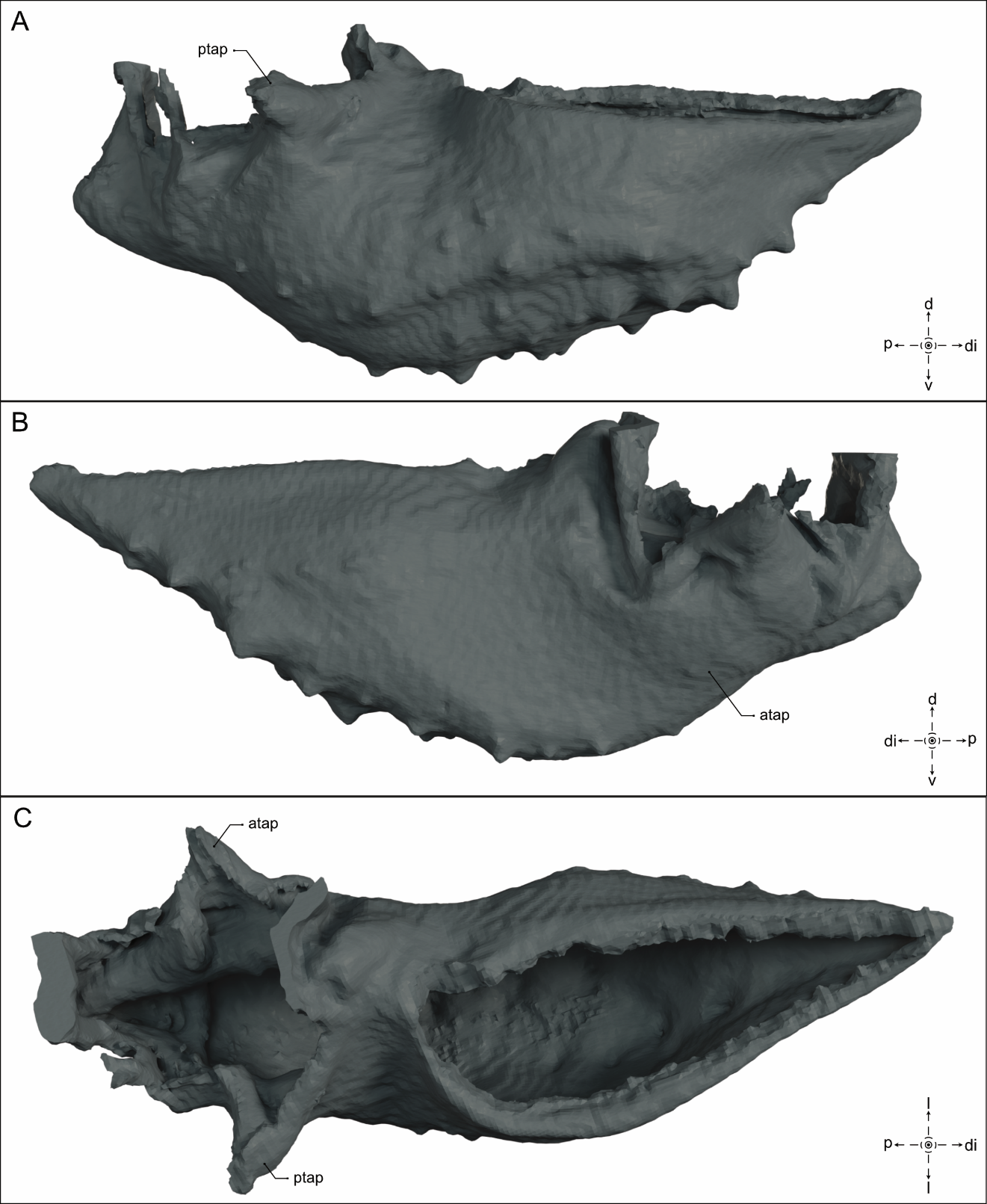


Figure S3 – Three-dimensional visualization of the trochanter from *µCT* data in posterior (A), anterior (B) and dorsal (C) view. An interactive 3D model of the trochanter can be found following this link: https://skfb.ly/pAOwA. Abbreviations: atap – anterior trochanteral articular protrusion; d – dorsal; di – distal; f – frontal; l – lateral; p – proximal; ptap – posterior trochanteral articular protrusion; v – ventral.


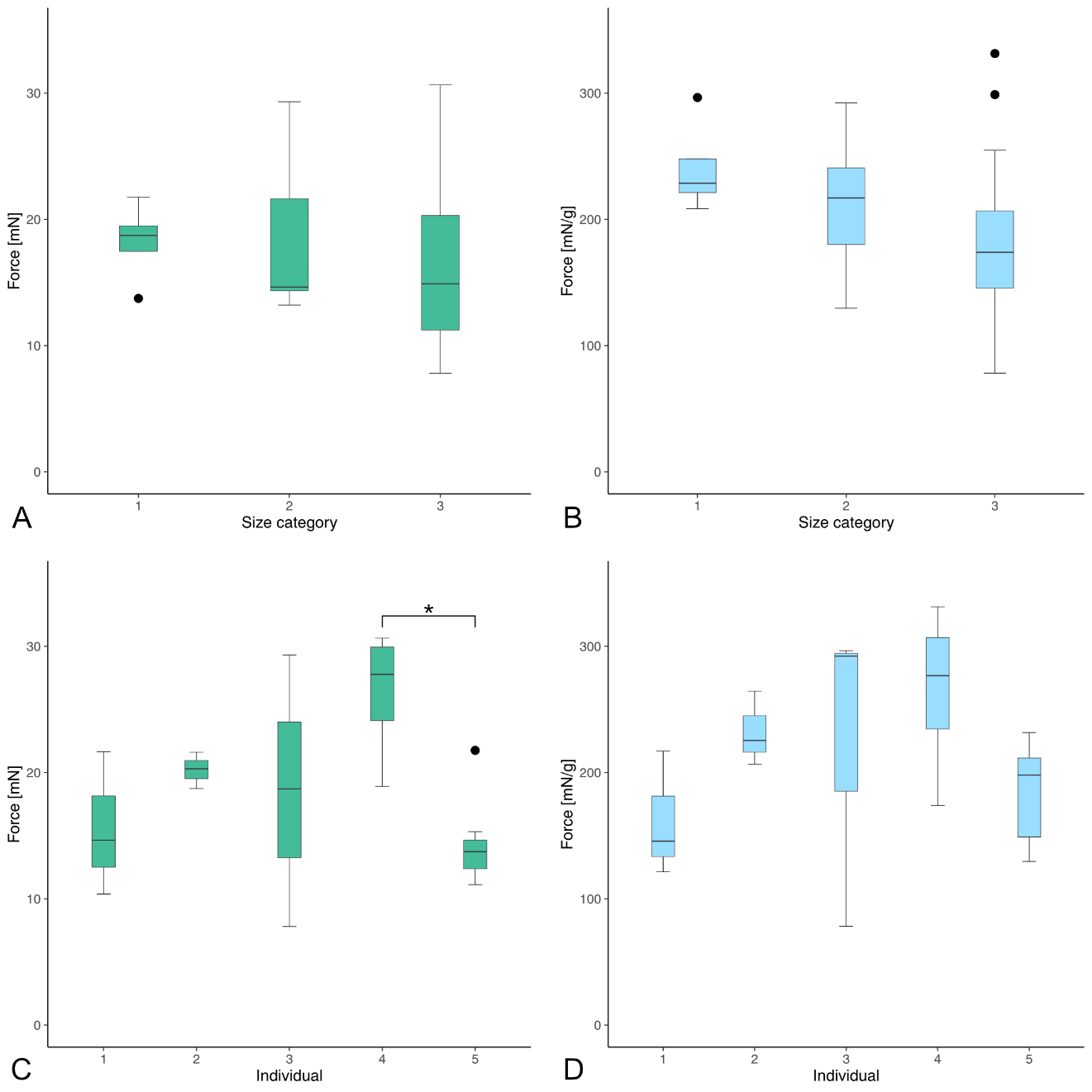
Figure S4 – Boxplots of the measured forces (A and C]) and forces per bodyweight (B and]) from force measurement experiments. A and B show the forces of all individuals pooled, against the different size categories of the prey items (1 – small, 2 – medium, 3 – large). C and D show the forces of all measured strikes for the individuals. *N* = 5; *n* = 3 for specimen 1, 2 and 3; *n* = 4 for specimen 4; *n* = 11 for specimen 5). The upper and lower borders of the boxes indicate the 25^th^ and 75^th^ percentiles, the line within the box represents the median and the whiskers indicate the 10^th^ and 90^th^ percentiles. Outliers are represented by black dots. The asterisk indicates statistically significant differences: * ≙ p < 0.05.


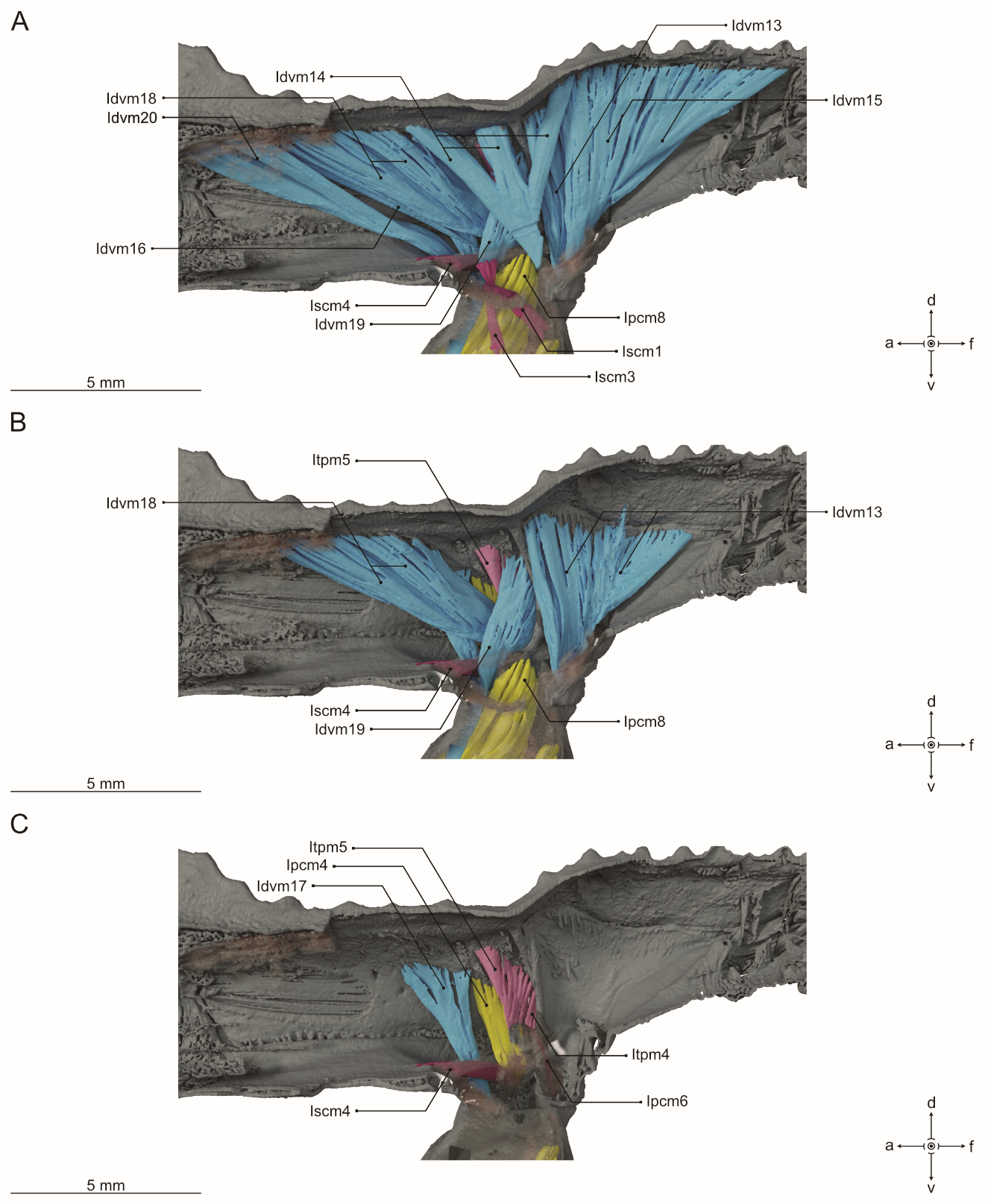
Figure S5 – Three-dimensional visualization of the extrinsic musculature of an adult female *Haania orlovi* from *µCT* data, medial view. In certain areas, the cuticle is displayed transparent or cutout, to allow for a better view of the musculature. Muscles are subsequently removed in every layer, to enable an understanding of the spatial arrangement. An interactive 3D model can be found following this links: https://skfb.ly/pAPwX, https://skfb.ly/pAPwY. A – first layer; B – second layer; C – third layer. Abbreviations: a – abdominal; d – dorsal; dvm – dorso-ventral muscle; f – frontal; pcm – pleuro-coxal muscle; scm – sterno-coxal muscle; v – ventral.


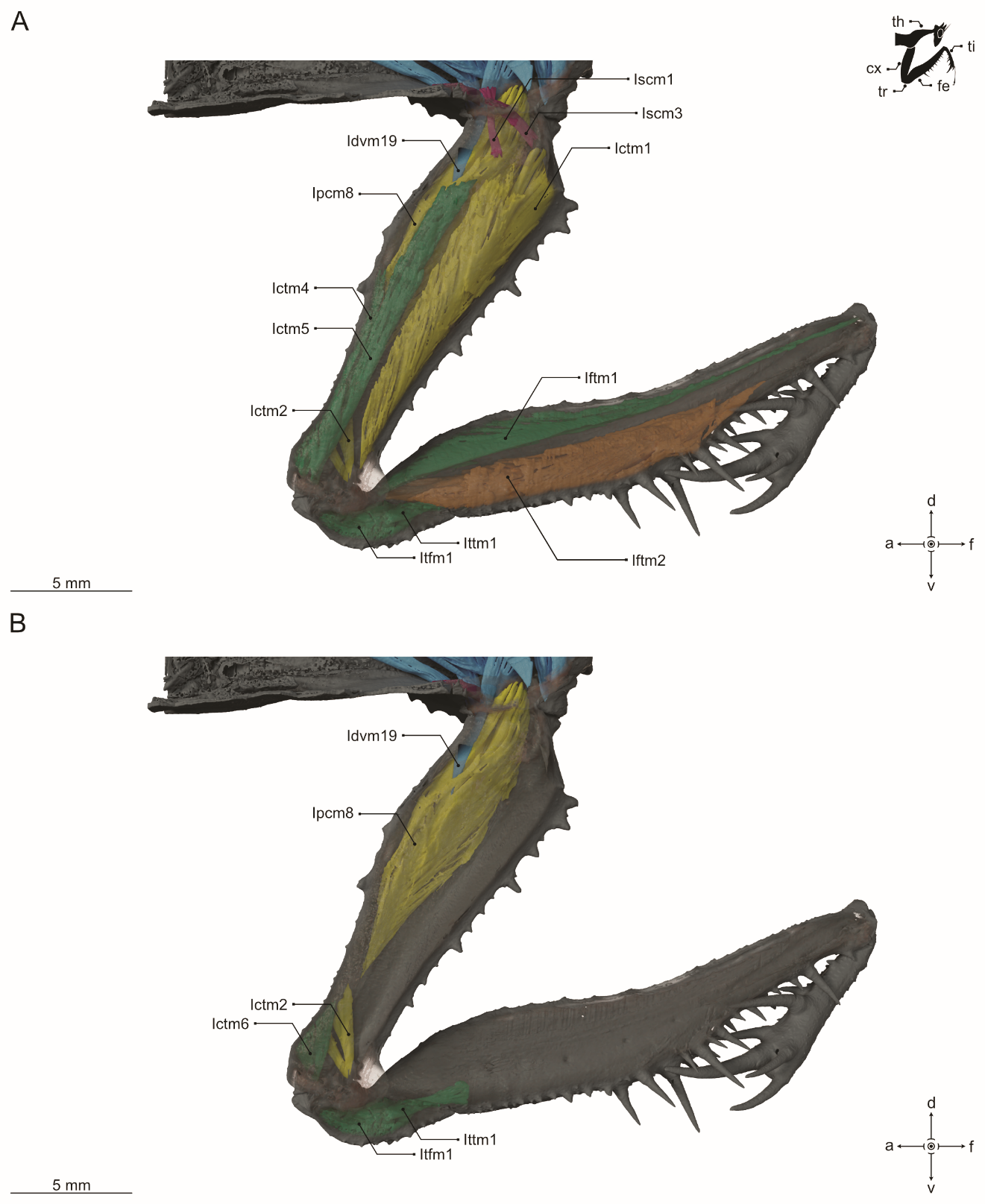
Figure S6 – Three-dimensional visualization of the intrinsic musculature of an adult female *Haania orlovi* from *µCT* data, medial view. In certain areas, the cuticle is displayed transparent or cut-out, to allow for a better view of the musculature. Muscles are subsequently removed in every layer, to enable an understanding of the spatial arrangement. The pictogram in the top right-hand corner shows a simplified overview of the cuticular parts of the animal. An interactive 3D model can be found following this link: https://skfb.ly/pAPwZ. A – first layer; B – second layer. Abbreviations: a – abdominal; cx – coxa; d – dorsal; dvm – dorso-ventral muscle; f – frontal; fe – femur; pcm – pleuro-coxal muscle; scm – sterno-coxal muscle; th – thorax; ti – tibia; tr – trochanter; v – ventral.

Table S1 – Kinematic data calculated from motion tracking analysis, used for calculations of the *mpo*. Abbreviations: AC – acceleration; AGL – angle; AVG – average; CT – coxa-trochanter joint; DIF – difference; FT – femur-tibia joint; MX – maximum; ST – start; V – velocity.

| ID | CT_ST_AGL [rad] | CT_MX_AGL [rad] | CT_DIF_AGL [rad] | FT_ST_AGL [rad] | FT_MX_ANL [rad] | FT_DIF_AGL [rad] | CT_MX_V [rads^-1^] | CT_AVG_V [rads^-1^] | FT_MX_V [rads^-1^] | FT_AVG_V [rads^-1^] | CT_MX_AC [rads^-2^] | CT_AVG_AC [rads^-2^] | FT_MX_AC [rads^-2^] | FT_AVG_AC [rads^-2^] |
| --- | --- | --- | --- | --- | --- | --- | --- | --- | --- | --- | --- | --- | --- | --- |
| 1 | 0.215 | 1.518 | 1.303 | 1.679 | 1.782 | 0.103 | 726.288 | 449.304 | 247.874 | 17.364 | 622478.359 | 338774.689 | 311849.009 | 40119.960 |
| 1 | 0.192 | 2.218 | 2.025 | 2.225 | 2.683 | 0.457 | 1878.590 | 972.229 | 745.619 | 144.044 | 1209414.616 | 919706.665 | 2803371.415 | 475133.018 |
| 1 | 0.184 | 2.139 | 1.955 | 2.150 | 2.484 | 0.335 | 2080.502 | 1019.898 | 834.186 | 146.636 | 1672950.911 | 1086892.596 | 2354972.667 | 424949.102 |
| 1 | 0.199 | 2.451 | 2.252 | 2.223 | 2.676 | 0.453 | 2471.226 | 1165.084 | 1740.661 | 211.486 | 2371443.449 | 1281692.156 | 4245850.767 | 684550.979 |
| 1 | 0.208 | 2.397 | 2.189 | 2.201 | 2.524 | 0.323 | 2203.749 | 1172.670 | 1128.157 | 146.031 | 3103367.212 | 1335848.707 | 3888581.470 | 490914.982 |
| 1 | 0.163 | 2.590 | 2.427 | 2.145 | 2.622 | 0.477 | 2261.383 | 1174.447 | 1213.491 | 135.662 | 3301659.333 | 1283424.271 | 6549204.872 | 619091.466 |
| 2 | 0.201 | 1.499 | 1.298 | 2.175 | 2.634 | 0.460 | 1769.320 | 846.655 | 942.284 | 164.223 | 1537267.936 | 1091173.976 | 4207376.217 | 762942.437 |
| 2 | 0.206 | 1.797 | 1.591 | 2.385 | 2.476 | 0.091 | 1865.671 | 954.671 | 521.665 | -56.944 | 1868625.608 | 1175157.713 | 1552282.375 | 99531.056 |
| 2 | 0.204 | 1.633 | 1.429 | 2.443 | 2.558 | 0.115 | 1491.537 | 824.523 | 480.617 | -58.820 | 1587499.931 | 975339.896 | 2038750.662 | 161108.631 |
| 2 | 0.198 | 1.574 | 1.376 | 2.184 | 2.326 | 0.142 | 1674.518 | 869.255 | 770.282 | -11.453 | 1338111.815 | 1027043.875 | 1837271.355 | 177290.409 |
| 2 | 0.176 | 1.739 | 1.563 | 2.376 | 2.552 | 0.176 | 1923.462 | 893.002 | 1402.111 | 61.372 | 1214817.844 | 971042.309 | 1561938.705 | 284309.977 |
| 3 | 0.196 | 2.005 | 1.809 | 1.903 | 1.930 | 0.026 | 1147.776 | 695.839 | 397.766 | -24.279 | 1336081.218 | 590675.828 | 1111718.679 | 21503.006 |
| 3 | 0.226 | 1.860 | 1.634 | 1.820 | 2.026 | 0.207 | 1047.121 | 612.652 | 512.229 | 39.079 | 612149.593 | 446166.460 | 1146535.377 | 112089.248 |
| 3 | 0.189 | 1.546 | 1.357 | 1.755 | 1.889 | 0.134 | 1120.301 | 656.847 | 502.944 | 47.899 | 1289992.240 | 722154.624 | 1886039.112 | 74429.303 |
| 4 | 0.196 | 1.803 | 1.607 | 1.705 | 1.956 | 0.251 | 1245.088 | 753.214 | 710.687 | 70.564 | 1370730.379 | 748936.684 | 2557280.846 | 257027.646 |
| 5 | 0.270 | 1.891 | 1.621 | 1.833 | 2.012 | 0.179 | 1293.767 | 784.208 | 391.105 | 44.661 | 1755284.060 | 824931.226 | 1695698.440 | 207790.799 |
| 5 | 0.227 | 2.123 | 1.895 | 1.945 | 2.231 | 0.286 | 2341.815 | 1093.371 | 863.426 | 162.397 | 1463634.625 | 1147455.982 | 2158564.363 | 249911.297 |
| 5 | 0.178 | 2.233 | 2.055 | 1.939 | 2.067 | 0.129 | 2389.842 | 1141.596 | 621.821 | -45.692 | 2170027.948 | 1240115.965 | 1534319.584 | 78590.278 |
| 6 | 0.213 | 1.505 | 1.292 | 1.708 | 1.808 | 0.100 | 733.007 | 416.901 | 197.830 | 21.462 | 360301.988 | 265204.976 | 293312.475 | 45092.901 |
| 6 | 0.260 | 1.832 | 1.571 | 1.762 | 1.961 | 0.199 | 1270.571 | 673.428 | 318.854 | 61.573 | 673817.557 | 504780.601 | 382624.986 | 128659.545 |
| 6 | 0.144 | 2.012 | 1.867 | 1.720 | 2.050 | 0.330 | 1532.041 | 829.989 | 452.239 | 146.731 | 843245.669 | 719537.659 | 1149756.598 | 248122.806 |
| 6 | 0.106 | 2.452 | 2.346 | 1.755 | 1.983 | 0.228 | 1395.243 | 853.034 | 540.803 | 47.371 | 1322899.686 | 655364.221 | 1102175.177 | 133055.825 |
| 7 | 0.226 | 2.116 | 1.891 | 1.919 | 2.356 | 0.437 | 1686.271 | 1012.839 | 729.858 | 208.843 | 1753292.984 | 1126544.043 | 2093760.329 | 437664.378 |
| 7 | 0.158 | 2.102 | 1.944 | 2.016 | 2.329 | 0.313 | 2065.548 | 1041.638 | 835.278 | 161.513 | 2551539.296 | 1185916.911 | 3890336.137 | 445784.557 |
| 8 | 0.214 | 2.076 | 1.862 | 1.762 | 1.913 | 0.151 | 1526.855 | 827.557 | 386.293 | 19.779 | 942698.937 | 725907.586 | 911926.796 | 138619.857 |
| 8 | 0.193 | 2.230 | 2.038 | 1.830 | 1.938 | 0.108 | 1395.454 | 843.119 | 442.233 | 7.954 | 961267.704 | 722155.907 | 884466.175 | 72282.396 |
| 8 | 0.206 | 2.155 | 1.949 | 1.664 | 1.932 | 0.268 | 1222.628 | 687.997 | 371.023 | 92.579 | 657278.222 | 487406.045 | 1181840.071 | 145209.445 |
| 8 | 0.194 | 2.276 | 2.082 | 1.633 | 1.940 | 0.308 | 1052.151 | 675.181 | 367.288 | 96.832 | 636859.460 | 459541.443 | 621870.399 | 150984.286 |
| 9 | 0.170 | 2.713 | 2.543 | 1.643 | 1.952 | 0.309 | 1611.817 | 924.719 | 544.126 | 112.309 | 937861.674 | 676537.370 | 824491.482 | 175973.427 |
| 9 | 0.180 | 2.108 | 1.928 | 1.819 | 2.094 | 0.275 | 1747.183 | 963.995 | 751.711 | 113.189 | 1269981.063 | 956060.588 | 1346331.906 | 256135.231 |
| 9 | 0.153 | 2.074 | 1.921 | 1.794 | 1.976 | 0.182 | 1443.032 | 823.333 | 556.227 | 78.060 | 1255867.437 | 822760.497 | 1591225.088 | 188264.049 |
| 10 | 0.200 | 1.984 | 1.784 | 1.722 | 1.917 | 0.195 | 1218.927 | 669.026 | 339.054 | 46.474 | 769273.023 | 519920.803 | 553255.117 | 105240.527 |
| 10 | 0.171 | 2.344 | 2.173 | 1.828 | 2.238 | 0.410 | 2006.178 | 1086.665 | 756.478 | 187.879 | 1354091.833 | 1073877.925 | 868040.893 | 273862.501 |
| 10 | 0.211 | 2.535 | 2.324 | 1.770 | 2.207 | 0.438 | 1667.214 | 996.009 | 1062.514 | 183.067 | 2183290.913 | 1047526.683 | 2464094.480 | 377855.082 |
| 10 | 0.197 | 2.145 | 1.948 | 1.854 | 2.307 | 0.453 | 1530.583 | 865.608 | 559.016 | 201.315 | 1929232.007 | 843998.342 | 3034434.054 | 354520.582 |
| 11 | 0.194 | 1.846 | 1.652 | 1.791 | 2.144 | 0.352 | 1531.450 | 861.716 | 839.685 | 183.870 | 2140359.655 | 949318.859 | 2956388.027 | 414367.064 |
| 11 | 0.226 | 1.920 | 1.695 | 1.886 | 2.047 | 0.161 | 1262.926 | 813.376 | 619.529 | 25.659 | 1409509.061 | 847031.417 | 2478114.895 | 188321.626 |
| 11 | 0.229 | 2.113 | 1.884 | 1.732 | 2.179 | 0.448 | 1520.585 | 904.405 | 392.065 | 201.598 | 1561272.501 | 930167.682 | 1597527.227 | 376621.747 |
| 12 | 0.194 | 1.873 | 1.680 | 1.922 | 2.118 | 0.197 | 1426.987 | 775.305 | 588.862 | 10.227 | 1671763.550 | 746940.287 | 1308891.733 | 173297.881 |
| 12 | 0.240 | 1.933 | 1.694 | 1.633 | 1.992 | 0.359 | 1382.914 | 769.879 | 376.682 | 108.742 | 890165.558 | 694724.574 | 1328427.230 | 255379.127 |
| 13 | 0.162 | 1.938 | 1.775 | 1.766 | 1.961 | 0.195 | 1335.707 | 819.429 | 648.542 | 10.715 | 1428093.162 | 794051.093 | 2594166.618 | 188059.647 |
| 13 | 0.199 | 2.019 | 1.820 | 1.825 | 2.064 | 0.238 | 1335.542 | 752.897 | 456.516 | 52.538 | 1298450.174 | 713238.160 | 2495134.009 | 247735.279 |
| 13 | 0.179 | 1.761 | 1.583 | 1.834 | 1.979 | 0.145 | 1050.144 | 610.407 | 338.171 | 11.033 | 567077.549 | 455909.775 | 608708.470 | 61406.705 |

Table S2 – List of all extrinsic and intrinsic muscles of the prothorax and the raptorial forelegs found in *Haania orlovi*. Generally, the attachment and insertion points present in *H. orlovi* are identical with the ones described in Bäumler et al. (7) for mantises with “standard shape” of the raptorial forelegs. If characteristics (e.g., points of attachment etc.) differ from previous descriptions, it is additionally mentioned in the characteristics column. Unfortunately, muscles of the tibia were not clearly definable, and are therefore labelled as uncertain Abbreviations: ctm – coxo-trochanteral muscle; dvm – dorso-ventral muscle; ftm – femoro-tibial muscle; pcm – pleuro-coxal muscle; scm – sterno-coxal muscle; tbm – tibio-basitarsal muscle; tpm – tergo-pleural muscle; tfm – trochantero-femoral muscle; tipm – tibio-praetarsal muscle; ttm – trochantero-tibial muscle; + – muscle is present; - – muscle not present; ? – presence uncertain.

| Names and Abbreviations | | Presence | Characteristics |
| --- | --- | --- | --- |
| Abbreviation following Bäumler et al. (7) | Name following Bäumler et al. (7) |  |  |
| Prothorax | | | |
| Extrinsic musculature | | | |
| Dorso-ventral | | | |
| *Idvm13* | M. pronoto-trochantinalis anterior | + |  |
| *Idvm14* | M. pronoto-trochantinalis posterior | + | Attaches with three prominent strands at the tergum |
| *Idvm15* | M. pronoto-trochantino-coxalis | + |  |
| *Idvm16* | M. pronoto-coxalis anterior | + |  |
| *Idvm17* | M. pronoto-coxalis posterior | + |  |
| *Idvm18* | M. pronoto-coxalis lateralis | + |  |
| *Idvm19* | M. pronoto-trochanteralis | + | No multiple strains at the attachment at the tergum |
| *Idvm20* | M. pronoto-coxalis medialis | + | Insertion together with Idvm17 |
| *Pleuro-coxal* | | | |
| *Ipcm4* | M. propleuro-coxalis superior | + |  |
| *Ipcm6* | M. propleuro-coxalis posterior | + | Comparably slim muscle |
| *Ipcm8* | M. propleuro-trochanteralis | + |  |
| *Sterno-coxalis* | | | |
| *Iscm1* | M. profurca-coxalis anterior | + |  |
| *Iscm2* | M. profurca-coxalis posterior | + |  |
| *Iscm3* | M. profurca-coxalis medialis | + |  |
| *Iscm6* | M. profurca-trochanteralis | - |  |
| *Tergo-pleural* | | | |
| *Itpm4* | M. pronoto-apodemalis anterior | + |  |
| *Itpm5* | M. pronoto-apodemalis posterior | + |  |
| Intrinsic musculature | | | |
| Coxa | | | |
| *Ictm1* | M. procoxa-trochanteralis-dorsalis anterior | + |  |
| *Ictm3* | M. procoxa-trochanteralis-dorsalis inferior | + |  |
| *Ictm4* | M. procoxa-trochanteralis-ventralis anterior | + |  |
| *Ictm5* | M. procoxa-trochanteralis extensor medialis | + |  |
| *Ictm6* | M. procoxa-trochanteralis-ventralis posterior | + |  |
| *Trochanter* | | | |
| *Itfm1* | M. protrochantero-femoralis | + |  |
| *Ittm1* | M. protrochantero-tibialis | + |  |
| *Femur* | | | |
| *Iftm1* | M. profemuro-tibialis dorsalis | + |  |
| *Iftm2* | M. profemuro-tibialis ventralis | + |  |
| *Ifpm1* | M. profemuro-pretarsalis | + |  |
| *Tibia* | | | |
| *Itipm1* | M. protibio-pretarsalis | ? |  |
| *Itbm1* | M. protibio-basitarsalis ventralis | ? |  |
| *Itbm2* | M. protibio-basitarsalis anterior | ? |  |
| *Itbm3* | M. protibio-basitarsalis posterior | ? |  |

Table S3 – List of all measured forces (Force [N]) and calculated maximum velocities (Max_Velocity [ms^-1^]) from experiments with artificial trochanters. Sample column shows the name of the sample, Friction shows the amount of friction in the locking structure (*H* = high, *L* = low), Type shows the type of artificial trochanter (*A* = non-deformable, *B* = deformable with a double spiral). Abbreviations: atrH – artificial trochanter hard; atrS – artificial trochanter spiral.

| Sample | Friction | Type | Force [N] | Max_Velocity [ms^-1^] |
| --- | --- | --- | --- | --- |
| *atrH1* | H | A | 44.44 | 0.0774 |
| *atrH1* | H | A | 46.88 | 0.0754 |
| *atrH1* | H | A | 46.17 | 0.0934 |
| *atrH1* | H | A | 44.98 | 0.0923 |
| *atrH1* | H | A | 46.55 | 0.0929 |
| *atrH1* | H | A | 47.84 | 0.1133 |
| *atrH1* | H | A | 38.18 | 0.0856 |
| *atrH1* | H | A | 38.86 | 0.0891 |
| *atrH2* | H | A | 16.43 | 0.0711 |
| *atrH2* | H | A | 14.86 | 0.0717 |
| *atrH2* | H | A | 14.83 | 0.0745 |
| *atrH2* | H | A | 14.19 | 0.0739 |
| *atrH2* | H | A | 13.55 | 0.0723 |
| *atrH2* | H | A | 13.99 | 0.0719 |
| *atrH2* | H | A | 13.64 | 0.0731 |
| *atrH2* | H | A | 13.38 | 0.0721 |
| *atrH3* | H | A | 22.67 | 0.0710 |
| *atrH3* | H | A | 20.22 | 0.0730 |
| *atrH3* | H | A | 20.5 | 0.0729 |
| *atrH3* | H | A | 19.9 | 0.0724 |
| *atrH3* | H | A | 19.84 | 0.0716 |
| *atrH3* | H | A | 17.79 | 0.0699 |
| *atrH3* | H | A | 17.07 | 0.0721 |
| *atrH3* | H | A | 16.99 | 0.0710 |
| *atrH4* | H | A | 15.34 | 0.0824 |
| *atrH4* | H | A | 13.22 | 0.0772 |
| *atrH4* | H | A | 12.42 | 0.0762 |
| *atrH4* | H | A | 11.1 | 0.0752 |
| *atrH4* | H | A | 11.22 | 0.0728 |
| *atrH4* | H | A | 10.86 | 0.0729 |
| *atrH4* | H | A | 9.27 | 0.0738 |
| *atrH4* | H | A | 9.07 | 0.0746 |
| *atrS1* | H | B | 20.08 | 0.2479 |
| *atrS1* | H | B | 21.68 | 0.2502 |
| *atrS1* | H | B | 20.21 | 0.2457 |
| *atrS1* | H | B | 21 | 0.2397 |
| *atrS1* | H | B | 22.03 | 0.2365 |
| *atrS1* | H | B | 17.08 | 0.2362 |
| *atrS1* | H | B | 17.1 | 0.2296 |
| *atrS1* | H | B | 18 | 0.2299 |
| *atrS2* | H | B | 2.95 | 0.0779 |
| *atrS2* | H | B | 2.75 | 0.0747 |
| *atrS2* | H | B | 4.46 | 0.1152 |
| *atrS2* | H | B | 3.69 | 0.0938 |
| *atrS2* | H | B | 2.97 | 0.0761 |
| *atrS2* | H | B | 4.45 | 0.1147 |
| *atrS2* | H | B | 2.78 | 0.0794 |
| *atrS2* | H | B | 3.42 | 0.0964 |
| *atrS3* | H | B | 33.73 | 0.2430 |
| *atrS3* | H | B | 31.3 | 0.2324 |
| *atrS3* | H | B | 36.36 | 0.2452 |
| *atrS3* | H | B | 35.42 | 0.2410 |
| *atrS3* | H | B | 26.67 | 0.2415 |
| *atrS3* | H | B | 29.97 | 0.2322 |
| *atrS3* | H | B | 30.24 | 0.2316 |
| *atrS3* | H | B | 30.99 | 0.2304 |
| *atrS4* | H | B | 21.91 | 0.2997 |
| *atrS4* | H | B | 19.83 | 0.2596 |
| *atrS4* | H | B | 19.26 | 0.2501 |
| *atrS4* | H | B | 23.1 | 0.2542 |
| *atrS4* | H | B | 18.69 | 0.2381 |
| *atrS4* | H | B | 15.7 | 0.2319 |
| *atrS4* | H | B | 19.31 | 0.2460 |
| *atrS4* | H | B | 19.07 | 0.2407 |
| *atrH1* | L | A | 2.18 | 0.0759 |
| *atrH1* | L | A | 0.08 | 0.0761 |
| *atrH1* | L | A | 0.07 | 0.0760 |
| *atrH1* | L | A | 0.1 | 0.0737 |
| *atrH1* | L | A | 0.09 | 0.0732 |
| *atrH1* | L | A | 0.09 | 0.0768 |
| *atrH2* | L | A | 0.3 | 0.0702 |
| *atrH2* | L | A | 0.22 | 0.0718 |
| *atrH2* | L | A | 0.3 | 0.0714 |
| *atrH2* | L | A | 0.34 | 0.0713 |
| *atrH2* | L | A | 0.7 | 0.0749 |
| *atrH2* | L | A | 0.62 | 0.0736 |
| *atrH2* | L | A | 0.39 | 0.0738 |
| *atrH2* | L | A | 0.51 | 0.0739 |
| *atrH3* | L | A | 0.41 | 0.0716 |
| *atrH3* | L | A | 0.4 | 0.0704 |
| *atrH3* | L | A | 0.39 | 0.0737 |
| *atrH3* | L | A | 1.68 | 0.0717 |
| *atrH3* | L | A | 0.37 | 0.0701 |
| *atrH3* | L | A | 0.31 | 0.0710 |
| *atrH3* | L | A | 0.27 | 0.0709 |
| *atrH3* | L | A | 0.93 | 0.0714 |
| *atrH4* | L | A | 0.79 | 0.0685 |
| *atrH4* | L | A | 0.85 | 0.0691 |
| *atrH4* | L | A | 0.77 | 0.0695 |
| *atrH4* | L | A | 0.76 | 0.0693 |
| *atrH4* | L | A | 0.79 | 0.0689 |
| *atrH4* | L | A | 0.73 | 0.0684 |
| *atrH4* | L | A | 0.68 | 0.0687 |
| *atrH4* | L | A | 0.49 | 0.0673 |
| *atrS1* | L | B | 0.3 | 0.0586 |
| *atrS1* | L | B | 0.28 | 0.0579 |
| *atrS1* | L | B | 0.25 | 0.0559 |
| *atrS1* | L | B | 0.36 | 0.0573 |
| *atrS1* | L | B | 0.22 | 0.0551 |
| *atrS1* | L | B | 0.49 | 0.0582 |
| *atrS1* | L | B | 0.21 | 0.0584 |
| *atrS1* | L | B | 0.09 | 0.0559 |
| *atrS2* | L | B | 0.14 | 0.0452 |
| *atrS2* | L | B | 0.06 | 0.0523 |
| *atrS2* | L | B | 0.04 | 0.0552 |
| *atrS2* | L | B | 0.04 | 0.0553 |
| *atrS2* | L | B | 0.04 | 0.0552 |
| *atrS2* | L | B | 0.05 | 0.0594 |
| *atrS2* | L | B | 0.04 | 0.0578 |
| *atrS2* | L | B | 0.04 | 0.0571 |
| *atrS3* | L | B | 0.68 | 0.0584 |
| *atrS3* | L | B | 0.6 | 0.0574 |
| *atrS3* | L | B | 0.53 | 0.0574 |
| *atrS3* | L | B | 0.49 | 0.0573 |
| *atrS3* | L | B | 0.61 | 0.0574 |
| *atrS3* | L | B | 0.37 | 0.0572 |
| *atrS3* | L | B | 0.61 | 0.0579 |
| *atrS3* | L | B | 0.56 | 0.0586 |
| *atrS4* | L | B | 0.38 | 0.0549 |
| *atrS4* | L | B | 0.39 | 0.0512 |
| *atrS4* | L | B | 0.43 | 0.0517 |
| *atrS4* | L | B | 0.37 | 0.0526 |
| *atrS4* | L | B | 0.84 | 0.0527 |
| *atrS4* | L | B | 1.05 | 0.0562 |
| *atrS4* | L | B | 0.85 | 0.0515 |
| *atrS4* | L | B | 0.94 | 0.0548 |

Table S4 – Data from force measurement experiments, high-speed videography and morphological analysis (*µCT*), associated with the calculation of the mass-specific power output. Prey sizes are categorized as small (S), medium (M) and large (L). For the calculations, the time for the duration of the movement (0.0027 s) and the angle (1.83 rad) was taken as the average of all analysed high-speed videos. The radius was calculated as described in the Methods S1. The muscle density for the calculations of the muscle mass for the actuating musculature in the coxa was varied to be the average of the literature value (1150 kgm^-3^) and the same value increased by 25% to account for possible shrinkage artifacts, as described in Methods S1.

| Animal | Prey size | Mass [g] | Power output [W] | Work [J] | Torque [Nm] | Force [N] | Radius [m] | Mass-specific power output [W/kg] literature density value | Mass-specific power output [W/kg] literature density value + 25% |
| --- | --- | --- | --- | --- | --- | --- | --- | --- | --- |
| 1 | M | 0.101 | 0.02289 | 6.1795exp-05 | 3.3768exp-05 | 0.0146 | 0.00231 | 5.1484exp+04 | 4.1187exp+04 |
| 1 | M | 0.100 | 0.03387 | 9.1437exp-05 | 4.9966exp-05 | 0.0217 | 0.00231 | 7.6180exp+04 | 6.0944exp+04 |
| 1 | L | 0.086 | 0.01623 | 4.3810exp-05 | 2.3940exp-05 | 0.0104 | 0.00231 | 3.6500exp+04 | 2.9200exp+04 |
| 2 | L | 0.098 | 0.03175 | 8.5716exp-05 | 4.6839exp-05 | 0.0203 | 0.00231 | 7.1413exp+04 | 5.7131exp+04 |
| 2 | M | 0.082 | 0.03379 | 9.1225exp-05 | 4.9850exp-05 | 0.0216 | 0.00231 | 7.6003exp+04 | 6.0803exp+04 |
| 2 | S | 0.083 | 0.02930 | 7.9101exp-05 | 4.3224exp-05 | 0.0187 | 0.00231 | 6.5902exp+04 | 5.2722exp+04 |
| 3 | L | 0.100 | 0.01220 | 3.2950exp-05 | 1.8006exp-05 | 0.0078 | 0.00231 | 2.7452exp+04 | 2.1962exp+04 |
| 3 | M | 0.100 | 0.04584 | 1.2377exp-04 | 6.7633exp-05 | 0.0293 | 0.00231 | 1.0312exp+05 | 8.2493exp+04 |
| 3 | S | 0.063 | 0.02925 | 7.8988exp-05 | 4.3163exp-05 | 0.0187 | 0.00231 | 6.5808exp+04 | 5.2647exp+04 |
| 4 | L | 0.093 | 0.04793 | 1.2940exp-04 | 7.0712exp-05 | 0.0306 | 0.00231 | 1.0781exp+05 | 8.6249exp+04 |
| 4 | L | 0.099 | 0.04644 | 1.2540exp-04 | 6.8523exp-05 | 0.0297 | 0.00231 | 1.0447exp+05 | 8.3579exp+04 |
| 4 | L | 0.102 | 0.04043 | 1.0915exp-04 | 5.9647exp-05 | 0.0259 | 0.00231 | 9.0940exp+04 | 7.2752exp+04 |
| 4 | L | 0.109 | 0.02956 | 7.9808exp-05 | 4.3611exp-05 | 0.0189 | 0.00231 | 6.6491exp+04 | 5.3193exp+04 |
| 5 | M | 0.067 | 0.02256 | 6.0913exp-05 | 3.3286exp-05 | 0.0144 | 0.00231 | 5.0749exp+04 | 4.0599exp+04 |
| 5 | L | 0.075 | 0.02395 | 6.4677exp-05 | 3.5343exp-05 | 0.0153 | 0.00231 | 5.3885exp+04 | 4.3108exp+04 |
| 5 | L | 0.083 | 0.01967 | 5.3110exp-05 | 2.9022exp-05 | 0.0126 | 0.00231 | 4.4249exp+04 | 3.5399exp+04 |
| 5 | L | 0.080 | 0.01739 | 4.6961exp-05 | 2.5662exp-05 | 0.0111 | 0.00231 | 3.9125exp+04 | 3.1300exp+04 |
| 5 | L | 0.075 | 0.02328 | 6.2857exp-05 | 3.4348exp-05 | 0.0149 | 0.00231 | 5.2369exp+04 | 4.1895exp+04 |
| 5 | M | 0.067 | 0.02231 | 6.0238exp-05 | 3.2917exp-05 | 0.0143 | 0.00231 | 5.0187exp+04 | 4.0150exp+04 |
| 5 | L | 0.076 | 0.01904 | 5.1409exp-05 | 2.8092exp-05 | 0.0122 | 0.00231 | 4.2831exp+04 | 3.4265exp+04 |
| 5 | L | 0.077 | 0.01756 | 4.7408exp-05 | 2.5906exp-05 | 0.0112 | 0.00231 | 3.9498exp+04 | 3.1598exp+04 |
| 5 | M | 0.102 | 0.02065 | 5.5755exp-05 | 3.0467exp-05 | 0.0132 | 0.00231 | 4.6452exp+04 | 3.7161exp+04 |
| 5 | S | 0.066 | 0.02148 | 5.7990exp-05 | 3.1688exp-05 | 0.0137 | 0.00231 | 4.8314exp+04 | 3.8651exp+04 |
| 5 | S | 0.094 | 0.03402 | 9.1861exp-05 | 5.0197exp-05 | 0.0218 | 0.00231 | 7.6533exp+04 | 6.1226exp+04 |

Table S5 – Data from calculations for the mass-specific power output of the second approach as described in Methods S1. For the calculations, the average angular velocity and angular acceleration of all analysed high-speed videos was used. The volume of the associated cuticle and musculature was calculated from µCT data and literature values for density, as described in the Methods S1. The muscle density for the calculations of the muscle mass for the actuating musculature in the coxa was varied to be the average of the literature value (1150 kgm^-3^) and the same value increased by 25% to account for possible shrinkage artifacts, as described in Methods S1.

| Inertia weighing method | Actuator muscle density [kgm^-3^] | Inertia muscle density [kgm^-3^] | Actuator muscle mass [kg] | Inertia [kgm²] | Average angular velocity [ms^-1^] | Average angular acceleration [ms^-2^] | Power output [W] | Mass-specific power output [Wkg^-1^] |
| --- | --- | --- | --- | --- | --- | --- | --- | --- |
| Scale | 1060 | - | 4.44547exp-07 | 2.3568exp-11 | 850.65 | 840254.82 | 0.01685 | 37893.59 |
| Scale | 1325 | - | 5.33457exp-07 | 2.3568exp-11 | 850.65 | 840254.82 | 0.01685 | 30314.87 |
| Calculated | 1060 | 4.19384exp-10 | 4.44547exp-07 | 1.91811exp-11 | 850.65 | 840254.82 | 0.01685 | 30840.13 |
| Calculated | 1060 | 5.03261exp-10 | 4.44547exp-07 | 2.05191exp-11 | 850.65 | 840254.82 | 0.01685 | 33529.42 |
| Calculated | 1325 | 4.19384exp-10 | 5.33457exp-07 | 1.91811exp-11 | 850.65 | 840254.82 | 0.01685 | 24672.1 |
| Calculated | 1325 | 5.03261exp-10 | 5.33457exp-07 | 2.05191exp-11 | 850.65 | 840254.82 | 0.01685 | 26823.53 |

Table S6 – Summary of the linear fixed-effects models (*LME*) for force (log-transformed) and velocity (original scale), that was used during analyses of the forces and velocities in the experiment with the artificial 3D-printed model.

| Response variable | Effect | Estimate | Standard Error | Degrees of freedom | Test statistic | p-value |
| --- | --- | --- | --- | --- | --- | --- |
| log(Force) | Intercept | 2.9605 | 0.3804 | 6.44 | t = 7.78 | 0.00017 |
| log(Force) | Type | −0.307 | 0.538 | 6.44 | t = −0.57 | 0.59 |
| log(Force) | Friction | −3.869 | 0.144 | 116 | t = −26.83 | <0.0001 |
| log(Force) | Type × Friction | −0.155 | 0.202 | 116 | t = −0.77 | 0.44 |
| Velocity | Intercept | 0.077 | 0.014 | 6.67 | t = 5.493 | 0.0011 |
| Velocity | Type | 0.128 | 0.020 | 6.67 | t = 6.40 | 0.00045 |
| Velocity | Friction | −0.005 | 0.0065 | 116 | t = −0.79 | 0.43 |
| Velocity | Type × Friction | −0.144 | 0.0092 | 116 | t = −15.73 | <0.0001 |

Table S7 – Results from the Type III analysis of variances for the fixed effects (Type, Friction, and Type x Friction), from the two *LME*s of force (log-transformed) and velocity (original scale).

| Response | Effect | Degrees of freedom | F-value | p-value |
| --- | --- | --- | --- | --- |
| log(Force) | Type | 1, 6.44 | 0.53 | 0.495 |
| log(Force) | Friction | 1, 116 | 1524.7 | <0.0001 |
| log(Force) | Type × Friction | 1, 116 | 0.59 | 0.445 |
| Velocity | Type | 1, 6.66 | 40.9 | 0.0289 |
| Velocity | Friction | 1, 116 | 0.62 | 0.43 |
| Velocity | Type × Friction | 1, 116 | 247.3 | <0.0001 |

**Table S8** – Results from within-Type pairwise contrasts**,** comparing the two friction scenarios high (*H*) and low (*L*), based on estimated marginal means, reported on the model’s scale (log scale for force, original scale for velocity)**.**

| Response | Type | Contrast | Estimate (Δ log) | Standard Error | Degrees of Freedom | 95% CI (log scale) | p-value |
| --- | --- | --- | --- | --- | --- | --- | --- |
| log(Force) | A | H − L | 3.87 | 0.144 | 116 | [3.58, 4.16] | <0.0001 |
| log(Force) | B | H − L | 4.02 | 0.142 | 116 | [3.74, 4.30] | <0.0001 |
| Velocity | A | H − L | 0.005 | 0.006 | 116 | [−0.008, 0.018] | 0.43 |
| Velocity | B | H − L | 0.149 | 0.006 | 116 | [0.137, 0.162] | <0.0001 |

Table S9 – Variance components for the random-effects, sample and residual, and derived intraclass correlation coefficients from the two *LME*s of force (log-transformed) and velocity (original scale).

| Response | Component | Variance | Standard Deviation | Intraclass correlation coefficient |
| --- | --- | --- | --- | --- |
| log(Force) | Sample (random intercept) | 0.5386 | 0.7339 | 0.63 |
| log(Force) | Residual | 0.3210 | 0.5666 | — |
| Velocity | Sample (random intercept) | 0.000713 | 0.0267 | 0.52 |
| Velocity | Residual | 0.000660 | 0.0257 | — |

Movie S1 (separate file) – High-speed video showing the predatory strike of an adult female *Haania orlovi* in lateral view with filmed 10 000 fps (individual images of important steps of the strike are displayed in Figure 2C-E in the main manuscript).

Movie S2 (separate file) – High-speed video showing the predatory strike of an adult female *Haania orlovi* in lateral view filmed with 20 000 fps with particular focus on the trochanter region (individual images of the deformation of the trochanter in Figure 2F, G, J and K in the main manuscript).

Movie S3 (separate file) – High-speed video showing video footage of an exemplary testing run of the artificial 3D-printed proof of concept of the *LaMSA* system in the high friction scenario filmed with 1000 fps in lateral view. The footage shows the sample *atrh1* without a deformable double spiral.

Movie S4 (separate file) – High-speed video showing video footage of an exemplary testing run of the artificial 3D-printed proof of concept of the *LaMSA* system in the high friction scenario filmed with 1000 fps in lateral view. The footage shows the sample *atrS1* with a deformable double spiral.

Movie S5 (separate file) – High-speed video showing video footage of an exemplary testing run of the artificial 3D-printed proof of concept of the *LaMSA* system in the low friction scenario filmed with 1000 fps in lateral view. The footage shows the sample *atrS3* with a deformable double spiral.

Movie S6 (separate file) – High-speed video showing video footage of an exemplary testing run of the artificial 3D-printed proof of concept of the *LaMSA* system in the low friction scenario filmed with 1000 fps in lateral view. The footage shows the sample *atrS1* with a deformable double spiral.

**References:**

1. Mendez J, Keys A. Density and composition of mammalian muscle. Metabolism. 1960;9:184–8.

2. Heimel P, Swiadek NV, Slezak P, Kerbl M, Schneider C, Nürnberger S, et al. Iodine-Enhanced Micro-CT Imaging of Soft Tissue on the Example of Peripheral Nerve Regeneration. Contrast Media Mol Imaging. 2019 Mar 27;2019:1–15.

3. Leonard KC, Worden N, Boettcher ML, Dickinson E, Hartstone‐Rose A. Effects of long‐term ethanol storage on muscle architecture. Anat Rec. 2022 Jan;305(1):184–98.

4. Singhal P. Evaluation of Histomorphometric Changes in Tissue Architecture in Relation to Alteration in Fixation Protocol – An Invitro Study. J Clin Diagn Res [Internet]. 2016 [cited 2025 Sept 4]; Available from: http://jcdr.net/article_fulltext.asp?issn=0973-709x&year=2016&volume=10&issue=8&page=ZC028&issn=0973-709x&id=8236

5. Vickerton P, Jarvis J, Jeffery N. Concentration‐dependent specimen shrinkage in iodine‐enhanced micro CT. J Anat. 2013 Aug;223(2):185–93.

6. Vincent JFV, Wegst UGK. Design and mechanical properties of insect cuticle. Arthropod Struct Dev. 2004 July;33(3):187–99.

7. Bäumler F, Gorb SN, Büsse S. Comparative Morphology of the Extrinsic and Intrinsic Leg Musculature in Dictyoptera (Insecta: Blattodea, Mantodea). J Morphol. 2024;285(12).
